# Age-dependent redox status in the brain stem of NO-deficient hypertensive rats

**DOI:** 10.1186/s12929-017-0366-4

**Published:** 2017-09-11

**Authors:** Miroslava Majzúnová, Zuzana Pakanová, Peter Kvasnička, Peter Bališ, Soňa Čačányiová, Ima Dovinová

**Affiliations:** 1 Institute of Normal and Pathological Physiology, Slovak Academy of Sciences, Sienkiewiczova 1, 813 71 Bratislava, Slovakia; 2 Institute of Chemistry, Slovak Academy of Sciences, Bratislava, Slovakia; 3 Institute of Particle and Nuclear Physics, Faculty of Mathematics and Physics, Charles University, Prague, Czech Republic

**Keywords:** NOS inhibition, Radical signaling, Antioxidant response, Brain stem

## Abstract

**Background:**

The brain stem contains important nuclei that control cardiovascular function via the sympathetic nervous system (SNS), which is strongly influenced by nitric oxide. Its biological activity is also largely determined by oxygen free radicals. Despite many experimental studies, the role of AT1R-NAD(P)H oxidase-superoxide pathway in NO-deficiency is not yet sufficiently clarified. We determined changes in free radical signaling and antioxidant and detoxification response in the brain stem of young and adult Wistar rats during chronic administration of exogenous NO inhibitors.

**Methods:**

Young (4 weeks) and adult (10 weeks) Wistar rats were treated with 7-nitroindazole (7-NI group, 10 mg/kg/day), a specific nNOS inhibitor, with N^G^-nitro-L-arginine-methyl ester (L-NAME group, 50 mg/kg/day), a nonspecific NOS inhibitor, and with drinking water (Control group) during 6 weeks. Systolic blood pressure was measured by non-invasive plethysmography. Expression of genes (AT1R, AT2R, p22phox, SOD and NOS isoforms, HO-1, MDR1a, housekeeper GAPDH) was identified by real-time PCR. NOS activity was detected by conversion of [3H]-L-arginine to [3H]-L-citrulline and SOD activity was measured using UV VIS spectroscopy.

**Results:**

We observed a blood pressure elevation and decrease in NOS activity only after L-NAME application in both age groups. Gene expression of nNOS (youngs) and eNOS (adults) in the brain stem decreased after both inhibitors. The radical signaling pathway triggered by AT1R and p22phox was elevated in L-NAME adults, but not in young rats. Moreover, L-NAME-induced NOS inhibition increased antioxidant response, as indicated by the observed elevation of mRNA SOD3, HO-1, AT2R and MDR1a in adult rats. 7-NI did not have a significant effect on AT1R-NADPH oxidase-superoxide pathway, yet it affected antioxidant response of mRNA expression of SOD1 and stimulated total activity of SOD in young rats and mRNA expression of AT2R in adult rats.

**Conclusion:**

Our results show that chronic NOS inhibition by two different NOS inhibitors has age-dependent effect on radical signaling and antioxidant/detoxificant response in Wistar rats. While 7-NI had neuroprotective effect in the brain stem of young Wistar rats, L-NAME- induced NOS inhibition evoked activation of AT1R-NAD(P)H oxidase pathway in adult Wistar rats. Triggering of the radical pathway was followed by activation of protective compensation mechanism at the gene expression level.

**Electronic supplementary material:**

The online version of this article (doi:10.1186/s12929-017-0366-4) contains supplementary material, which is available to authorized users.

## Background

The sympathetic nervous system (SNS) is one of the autonomic nervous pathways with a dominant role in the regulation of short- and long-term blood pressure. SNS regulates heart rate, contractility (systolic volume) and vasoconstriction on the periphery through adrenergic receptors [1]. Experimental and clinical evidence indicates that activity of the SNS increases in hypertension and abnormal activity of the sympathetic vasomotor tone is one of the factors responsible for the development of various forms of hypertension [2].

The SNS activity is strongly influenced by nitric oxide (NO) produced in the nuclei of the brain stem: nucleus tractus solitarii (NTS), and rostral ventrolateral medulla (RVLM) [3].

NO is synthesized from L-arginine through the constitutive Ca^2+^- dependent neuronal NOS (nNOS, NOS1) primarily expressed in neurons and glial cells, endothelial NOS (eNOS, NOS3) present in endothelium, platelets and cardiomyocytes (both in low nanomolar concentrations) [4]. Inducible NOS (iNOS, NOS2) is localized in macrophages, smooth muscle cells and glial cells, and is produced in micromolar levels) [5, 6].

Physiological regulation of vasomotor outflow by the endogenous NO at the RVLM is determined by a balance between sympathoexcitation and sympathoinhibition. The regulation operates on the tonically active nNOS and iNOS [7], with minimal contribution from eNOS in acute experiments [8]. Furthermore, whereas nNOS and iNOS are present in RVLM neurons, eNOS is associated primarily with blood vessels [8, 9].

NO deficiency and/or NOS inhibition play an important role in the development of hypertension. In most experiments, NO deficiency has been achieved by N^G^-nitro-L-arginine methyl esther (L-NAME) administration. Although L-NAME is not a specific NO synthase inhibitor, it assumed to preferentially inhibit eNOS [10]. Chronic administration of L-NAME leads to development of persistent hypertension related to peripheral vasoconstriction and increased peripheral resistance of vessels [11]. Increased activity of the SNS was observed in L-NAME-induced hypertension [12]. The effect of systemic nNOS inhibition on the cardiovascular system has not been extensively studied.

It was observed that chronic administration of the 7-NI nNOS blocker to SHR rats altered calcium handling and regulation of various metabolic pathways in kidney [13]. Wang et al. confirmed that 7-NI in adult SD rats crosses the blood brain barrier [14]. The inhibition of nNOS with the specific inhibitor 7-nitroindazole (7-NI) for several days and/or weeks does not significantly affect the blood pressure of normotensive [15–18] and spontaneously hypertensive rats [19], but blood pressure-independent hypotrophy of the heart, kidney and arterial walls of conduit arteries was observed in normotensive Wistar rats [18, 20].

Neuronal NOS-produced NO has a variety of effects in the CNS and one of this is stimulatory and inhibitory influence of sympathetic activity observed in rats [21, 22]. In the RVLM of the metabolic syndrome rats, nNOS uncoupling was observed where the ratio of nNOS dimer/monomer was significantly decreased [23].

Increased levels of oxidative stress are observed in the brain of hypertensive models of rats. This can lead to changes in the sympathetic vasomotor tone and the development of hypertension [24, 25]. In the RVLM of spontaneously hypertensive rats, oxidative stress is chronically elevated due to impairment of the mitochondrial electron transport chain and reduced activity of antioxidant (superoxide dismutase/catalase), resulting in neurogenic hypertension [26]. Stroke-prone spontaneously hypertensive rats have oxidative stress increased in the whole brain in contrast with normotensive control Wistar-Kyoto rats [27]. In addition, elevated O_2_- may contribute to hypertension by reducing the NO-promoted cardiovascular depression [28]. Bioavailability of NO is regulated by ROS levels and SOD activity. ROS and NO can form a highly reactive intermediate, peroxynitrite (ONOO-), which is cytotoxic in high concentrations and can cause oxidative damage to proteins, lipids and DNA [29]. In addition, ROS can lead to uncoupling of eNOS and nNOS and production of more superoxide [23, 30].

Important sources of ROS are NAD(P)H oxidase (Nox) isoforms in the brain, which are activated by angiotensin II (Ang II) via angiotensin 1 receptors (AT1R) [31, 32]. There is good evidence that in particular Nox2 and Nox4 are involved in the regulation of blood pressure through the brain renin-angiotensin system [33–35]. Besides Nox, Ang II stimulates production of ROS in mitochondria, which attenuates activity of baroreceptors and increases stimulation of the SNS [36–38]. While the AT1R in the CNS is linked to sympatho-excitation, activation of angiotensin 2 receptors (AT2R) exhibits opposite influence on sympathetic tone. Intra-cerebro-ventricular or intrarenal application of AT2R-agonists reduces blood pressure, but systemic application does not [39]. Gao and Zucker observed AT2Rs decrease blood pressure via a nNOS/NO signaling pathway within paraventricular nucleus and RVLM in normal rats [40]. Ex vivo and in vivo studies revealed that application of antagonist AT2R does not affect blood pressure, but significantly reduce collagen accumulation within the vascular wall and thereby also vascular stiffness. Despite the lack of antihypertensive effect in most instances, AT2R-stimulation is still able to attenuate hypertensive end-organ damage in kidneys, vasculature and the brain [39, 41, 42].

The presence of increased oxidative stress leads to stimulation of antioxidant response to re-establish the balance of redox state. Superoxide dismutases (SODs) represent the first line of antioxidant defense system. There are three SOD superoxide isoforms: copper-zinc SOD (Cu/ZnSOD, SOD1) located in cytosol, mitochondrial manganese SOD (MnSOD, SOD2), and extracellular SOD (ecSOD, SOD3) present in extracellular space [43]. The expression and activity of the ROS degradative enzymes, particularly SOD and catalase, are notably reduced in the RVLM of hypertensive animals. Administration of Tempol (SOD mimetic) or overexpression of SOD or catalase in RVLM decreases superoxide (O_2_^∸^) and H_2_O_2_ in brain, leading to reduction in sympathetic vasomotor activity in hypertensive animals [28, 44]. In vivo study shows that adenoviral vectors encoding SOD1 prevent superoxide production from Ang II infusion and the onset of hypertension [45]. Treatment with the mitochondrial targeted antioxidant mitoTEMPO decreased mitochondrial O_2_^∸^, inhibited the total cellular O_2_^∸^, reduced cellular NAD(P)H oxidase activity and restored the level of NO bioavailability. These effects were mimicked by overexpressing the mitochondrial MnSOD, while MnSOD depletion with siRNA increased both basal and Ang II-stimulated cellular O_2_^∸^ [46]. High levels of ecSOD in the extracellular matrix of arteries prevents transfer of NO from endothelial cells to smooth muscle cells. An immunostaining study established rare presence of ecSOD in the brain stem with only occasional cells observed in the central segmentum [47].

The Nrf2/Keap1/ARE (nuclear factor-E2-related factor/Kelch-like ECH-associated protein 1/antioxidant response element) signaling pathway is an important regulator of cellular resistance to oxidants and electrophiles. Transcription factor Nrf2 stimulates phase II of detoxification and antioxidant genes (e.g. SODs, hemeoxygenase-1 (HO-1), catalase, glutathione peroxidase) [48]. Expression of HO-1 has potential hypotensive effects and its upregulation has been observed in spontaneously hypertensive rats [49].

The permeability glycoprotein (P-gp) is an important protein transporter in the blood brain barrier (BBB). It is an encoded product of the human multidrug resistance (*MDR1*) gene, with a broad substrate specificity, including a variety of structurally divergent drugs in clinical use today [50–52]. In rodents, the multidrug resistance type I Pgp is encoded by two genes (*MDR1a*, *MDR1b*), and only *MDR1a* is localized in rodent brain capillaries. P-gp mediates the export of drugs from cells located in the gastrointestinal tract, hepatocytes, kidney proximal tubules and the blood-brain barrier, where it limits the entry of many drugs to the CNS [50, 53]. Wagner et al. (1997) observed a large increase in cerebral blood flow (CBF) in the hemispheres, brain stem, cerebellum, thalamus, and white matter after fluorocarbon (FC)-exchange transfusion in cats. They have shown that l-NAME inhibits brain NOS activity in FC-perfused cats, but does not reverse FC-exchange transfusion-induced CBF [54]. Kaufmann et al. (2004) [55] assessed the effect of simultaneous inhibition of eNOS and nNOS on myocardial blood flow (MBF) and coronary flow reserve (CFR) in volunteers and in (denervated) transplant recipients. They used nonspecific exogenous NO-inhibitors, L-NMMA (N(G)-monomethyl-L-arginine), L-NAME and endogenous ADMA [56]. It was found that intravenous infusions of L-NMMA (3 and 10 mg/kg) crosses the blood-brain barrier and inhibits eNOS and nNOS [55]. Stases, BBB disturbances and initial microvascular dysfunction has been observed in SHRSP animals and BBB damage was observed in these animals already at young age [57]. Biancardi et al. have confirmed sympathetic activation in rats with L-NAME-induced hypertension, where the hemodynamic pattern and the contribution of the sympathetic nervous system was studied in Wistar rats using oral gavage of L-NAME (20 mg/kg daily). The study shows that the vasoconstriction in response to L-NAME was mediated by the sympathetic drive [58], which plays an important role in the initiation and maintenance of hypertension.

The aim of our experiments was to determine changes in free radical signaling, antioxidant and detoxification response in the brain stem using chronic systemic administration of exogenous NOS inhibitors. We compared responses in young and adult Wistar rats after chronic NOS inhibition using L-NAME or 7-NI. We compared changes in eNOS and nNOS, in the stimulation of the AT1R-NAD(P)H oxidase pathway, in the antioxidant and detoxification defense system and in MDR1a involved in the BBB.

## Methods

### Animal models

We used male young (age 4 weeks) and adult (age 10 weeks) Wistar rats. Young and adult rats were divided into three groups by the type of administered compounds. The first group of youngs was treated with 7-nitroindazole (7-NI, Sigma) diluted in drinking water in the dose of 10 mg/kg/day (*n* = 7). The second group of youngs was treated with N^G^–nitro L–arginine methyl esther (L-NAME, Sigma) diluted in drinking water in the dose of 50 mg/kg/day (*n* = 7). The third group of young rats was the control group with pure drinking water (*n* = 7). The adult rats received the same treatment with 7-NI (*n* = 6), L-NAME (*n* = 5) and control groups (*n* = 6) as the young rats.

Both substances, 7-NI and L-NAME, were administered in young and adult rats continuously during 6 weeks. Body weight of rats, daily consumption of food and water were observed during the whole treatment period. Animals were placed in an air conditioned room at a constant temperature (24 °C) and humidity (45–60%) with a light regime of 12:12 h light / dark cycle (light phase from 6.00 to 18.00). They were fed standard pellet for rats and drinking water ad libitum. All animal experiments were performed in accordance the rules of the State Veterinary and Food Administration of the Slovak Republic and in accordance with the Institutional guidelines of the Slovak Academy of Sciences issued by its Animal Research and Care Committee.

### Measurement of blood pressure

Systolic blood pressure was measured by non-invasive plethysmographic method on the tail through Statham Pressure Transducer P23XL (Hugo Sachs, Germany) in all groups of rats. Blood pressure was observed every week at the same time during the whole period of experiment.

### Sample preparation

After long-term therapy, rats were sacrificed. Brain stem was quickly extracted and stored for further measurements depending on the method used later. Tissues for activity determination of nitric oxide synthases and superoxide dismutases were stored in an ice Tris-HCl with the addition of protease inhibitors.The remaining samples were rapidly frozen in liquid nitrogen and stored at −80 °C until use. The amount of proteins was determined by Lowry method.

#### Cell culturing of the SH-SY5Y neuronal cell line

Neuroblastoma cell lines SH- SY5Y (obtained from the ATCC) has been cultured in the mixture of Minimum Essential Medium (MEM, Sigma) and F − 12 Ham’s medium (Sigma) in 1:1 ratio with the addition of 1% of glutamine, penicillin-streptomycin solution, NEAA – nonesencial aminoacids and 10% fetal bovine serum (Sigma). The cell culture was cultivated in a CO_2_ incubator at 37°С and 5% CO_2_. Cells were subcultured every 5–7 days. The medium was changed every 3 days.

### Determination of NOS activity

Activity of NOS was measured by conversion of radioactive [3H]-L-Arginine (Amersham, UK) to 3H–L-Citrulline [59] with small modifications [60]. Activity of NOS was measured in 20% homogenates in Tris-HCl with the addition of protease inhibitors. The homogenates was centrifuged at 5000 rpm, 10 min at 4 °C. Samples were measured in duplets. The reaction mixture (consists of 10 mM NADPH; 0.5 M Tris, pH 7.4; 20 mM CaCl_2_ (MgCl_2_); 100 μM L-Arginine; 1 mg/ml calmodulin; FAD/FMN 1:1; radio-labeled L-arginine; 50 mM BH4; distilled water) and homogenates (50 μl) were incubated 6 min at 37 °C. After incubation, the reaction was started by addition of reaction mixture (50 μl) to samples. The reaction was stopped by solution with 0.02 M HEPES, 2 mM EDTA, 2 mM EGTA a 1 mM L-citrulline after 20 min. 1 ml from sample was applied to Dowex column in Na^+^ cycle with 1.5 ml distilled water. Subsequently, the product (samples with scintillation fluid ECOLIT) was detected on the Tri-Carb 2910 TR (Perkin Elmer) scintillation counter. NOS activity was expressed as pkat/g of proteins.

#### Fluorescent detection of NO production using the fluorescent dye DAF –FM diacetate in the SH-SY5Y neuroblastoma cell line

Indicator for nitric oxide determination: 4,5-diaminofluorescein diacetate (DAF-FM diacetate, Molecular Probes). The non-fluorescent dye is pass across cell membrane. The cell’s estherase activity turns the non fluorescent dye into a weakly fluorescent form and nitric oxide binds to this intracellular dye and increases the fluorescence activity in cells. The effect of NO inhibitors (7-NI and L-NAME) on NO production in the SH-SY5Y cell model was detected using an inverted fluorescence microscope (NIKON Eclipse Ti-E) and the NIS Elements AR program. The neuroblastoma cells of the SH-SY5Y line containing PgP and MRP proteins were seeded into 24-well plates, with 6 × 10^4^ cells per well and incubated for 3 days. After seeding, the cells were treated with NO inhibitors L- NAME (100uM) and 7-NI (50uM) during 24 h. After treatment, the cells were stained with 10uM DAF-FM diacetate (30 min in 37 °C in CO_2_ incubator), rewashed by DMEM buffer (without FBS and phenol red) and measured by fluorescence miscroscope exc/em 488/510 nm. Image J program was used for image analysis.

### Measurement of SOD activity

Total SOD activity was analyzed by the SOD Assay Kit (Fluka) in 0.5% homogenates in Tris-HCl with the addition of protease inhibitors according to the manufacturer’s protocol. Activity of SOD was measured as inhibition of production of formazan (WST-1-formazan) from tetrazolium salt (WST-1), which reacts with superoxide anions. Samples were incubated 20 min at 37 °C. Absorbance was measured on a spectrophotometer (Thermo Scientific Multiskan FC) at 450 nm. The resulting values were calculated using a standard curve and expressed as U / mg (Unit / milligram) of protein.

### Measurement of gene expression by quantitative PCR (qPCR)

The total RNA from the brain stem samples (50–100 was isolated by TRIsure reagent (Bioline) according to the manufacturer’s protocol. Isolated total RNA was quantified spectrophotometrically at 260/280 nm on Nanodrop 2000c (ThermoSci). Purity of the RNA was evaluated by the ratio between the absorbance values at 260 nm and 280 nm (A260 / A280). We worked with the pure RNA with the ratio of the optical densities in the range 1.9 to 2.1. TetrocDNA Synthesis kit (Bioline) was used for the reverse transcription and SensiFAST SYBRNoROX kit (Bioline) was used for the real-time polymerase chain reaction (PCR) according to the manufacturer’s protocol. Amplification of the cDNA was performed on a BioRad CFX96 Realtime system. All primers (100 pmol/ul) used for amplification of the studied genes (SOD1, SOD2, SOD3, NOS1, NOS3, p22phox, AT1R, AT2R, HO-1, MDR1a and GAPDH) were used according to Dovinova et al. [61]. Primer sequences (5′ to 3′) for HO-1, AT2R and MDR1a were as follows (forward and reverse, respectively): HO-1 (CAG GCA TAT ACC CGC TAC CT and TCT GTC ACC CTG TGC TTG AC); AT2R (GCC TGC ATT TTA AGG AGT GC and ACT GCT GGT GAT GTT TCT GCT) and MDR1a (TGT AAG CAG AAA GGT GTG GTA TGT and TCA TAG TGT TTC AGT ACG GCA TTT). The glyceraldehyde-3-phosphate dehydrogenase (GAPDH) was used as “housekeeping” gene.

### Statistical analysis

Data of adult and young animals were processed separately. The gene expression data for a whole set of genes were processed by fitting a 3-factor linear model (3- way ANOVA) using a script written in R [62]. The model was of the form ln(C / C ref) = μ 0 + μ DONOR + μ GENE, GROUP. A small number of outlying points were detected using the get Outliers method of the R’s *extremevalues* package [63], with default parameter settings. The outliers were removed from the dataset. This lead to removal of ~4% of values and to a distribution of residuals close to homoscedastic normal. Next we used the *glht* method from the R’s multcomp package [64] to calculate t-statistics for between-group differences. Adjusted *p*-values were calculated using the Westfall-Young maxT free step-down permutation algorithm to account for the large number of comparisons, correlations among comparisons and slightly non-gaussian distribution of model residuals [65].

## Results

### Effect of NOS inhibitors on systolic blood pressure

Only chronic administration of L-NAME changed systolic blood pressure in young and adult Wistar rats. Blood pressure was increased in L-NAME experimental groups at the end of experiments. Inhibition of neuronal NOS by 7-NI did not invoke this increase in any group (Table 1). In young rats chronic treatment with L-NAME after 6-weeks affected borderline hypertension 20.7% increase of blood pressure), while in adult rats high blood pressure increase was observed (54.3%).

**Table 1:** Systolic blood pressure changes in Wistar rats after chronic administration of 7-NI and L-NAME

	Young rats			Adult rats		
	Control (mmHg)	7-NI (mmHg)	L-NAME (mmHg)	Control (mmHg)	7-NI (mmHg)	L-NAME (mmHg)
1.week	102 ± 3	105 ± 3	107 ± 7	113 ± 5	114 ± 4	111 ± 4
3.week	106 ± 3	106 ± 2	120 ± 5**	114 ± 3	114 ± 3	163 ± 6**
6.week	115 ± 3	122 ± 6	139 ± 7**	110 ± 4	112 ± 9	166 ± 4**

Data represent mean ± std. dev., **P < 0.01; L-NAME, 7-NI vs. control

### Effect of NOS inhibitors on gene expression of eNOS and nNOS in brain stem

**Fig. 1:**
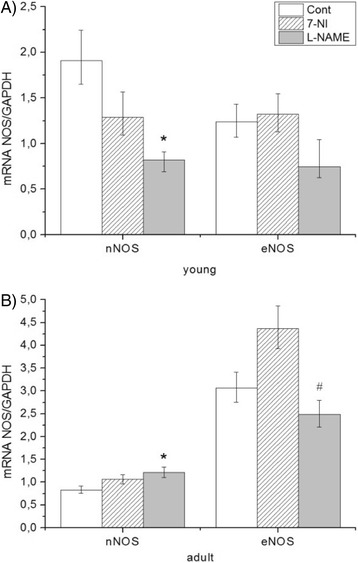
Effect of NOS inhibitors on NOS isoforms in young (**a**) and adult (**b**) Wistar rats. Expression of both genes was normalized on “housekeeping gene” GAPDH in brain stem. Data represent mean ± std. dev. *P < 0.05, L-NAME or 7-NI versus control. 7-NI (7-nitroindazole), L-NAME (N^G^ – nitro – L – arginine - methyl esther), GAPDH (glyceraldehyde-3-phosphate dehydrogenase), eNOS (endothelial NOS), nNOS (neuronal NOS)

Gene expression of mRNA(messenger RNA) nNOS was decreased in young Wistar rats after administration of 7-NI and L-NAME. On the contrary, significant increase was observed in adults after L-NAME treatment. We observed no changes after 7-NI treatment. On the other hand, we observed changes in gene expression of mRNA eNOS only in adults, where expression significantly declines after both types of inhibitors (Fig. 1b).

### Radical signaling: Effect of NOS inhibitors on AT1-NAD(P)H oxidase pathway and AT2R in the brain stem

**Fig. 2:**
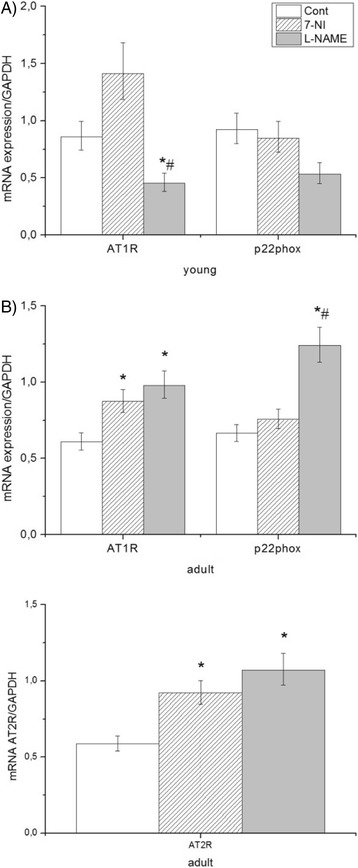
Effect of NOS inhibitors on AT1-NAD(P)H pathway in young (**a**) and adult (**b**) Wistar rats. Expression of mRNA of AT1R and, p22phox subunits of NAD(P)H oxidase was normalized on expression of GAPDH in brain stem. Data represent mean ± std. dev., *P < 0.05, L-NAME vs. control, #*P* < 0.05, 7-NI vs. L-NAME. 7-NI (7-nitroindazole), L-NAME (N^G^- nitro–L–arginine methyl esther), GAPDH (glyceraldehyde-3-phosphate dehydrogenase), AT1R (angiotensin receptor 1)

We observed that mRNA AT1R was overexpressed in adult rats treated with L-NAME (Fig. 2b), while the opposite was observed in young animals (Fig. 2a). The final increase of p22phox subunit of NAD(P)H oxidase was only in adult L-NAME animals and this growth was significant also comparing with the 7-NI group (Fig. 2b). These results suggest that administration of L-NAME stimulates radical signaling and production of free radicals through NAD(P)H oxidase via AT1R in the brain stem of adult Wistar rats. Treatment with 7-NI did not cause any statistically significant changes dependent on measured parameters (genes and age). However, there is a unconfirmed decrease in young animals and increase in adults in the expression of mRNA AT1R and p22phox (Fig. 2a, b).

The compensative effect of AT2R was also observed in adult rats. We found a significant increase of this gene in L-NAME (63.5%) and 7-NI (57%) group.

There was no age-dependent effect on mRNA of AT1R/NADPH oxidase (Table 2).

**Table 2:** The age-dependent effect on AT1R/NADPH oxidase and the redox status in Wistar rats

Gene	Young rats	Adult rats
mRNA AT1R/GAPDH	0,859 ± 0,1	0,610 ± 0,08
mRNA p22phox/GAPDH	0,923 ± 0,1	0,665 ± 0,08
mRNA SOD1/GAPDH	3096 ± 0,38	3136 ± 0,38
mRNA SOD2/GAPDH	0,475 ± 0,06	3302 ± 0,4**
mRNA SOD3/GAPDH	1272 ± 0,14	0,701 ± 0,09**

Comparison of mRNA levels of young and adult rats at native groups. Data represent mean ± std. dev., **P < 0,01; adult control vs. young control

Expression of mRNA of AT1R and, p22phox subunits of NAD(P)H oxidase was normalized on expression of GAPDH in brain stem. Data represent mean ± std. dev., *P < 0.05, L-NAME vs. control, #*P* < 0.05, 7-NI vs. L-NAME. 7-NI (7-nitroindazole), L-NAME (N^G^- nitro–L–arginine methyl esther), GAPDH (glyceraldehyde-3-phosphate dehydrogenase), AT1R (angiotensin receptor 1).

### Antioxidant and detoxification responses: Effect of inhibitors of NOS on antioxidant response, Nrf2 activation and multidrug resistance in the brain stem

**Fig. 3:**
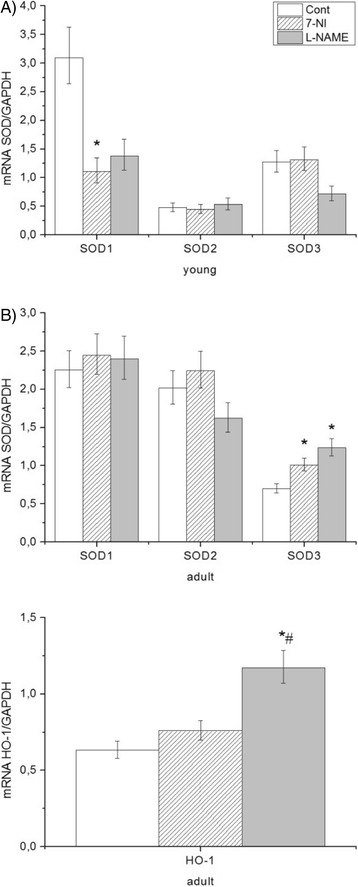
Effect of NOS inhibitors on expression of SOD isoforms in young (**a**) and adult (**b**) Wistar rats. Expression of individual SOD isoforms were normalized to expression of GAPDH in the brain stem . Data represent mean ± std. dev.. *P < 0.05, 7-NI or L-NAME vs. control; #*P* < 0.05, 7-NI vs. L-NAME. 7-NI (7-nitroindazole), L-NAME (N^G^–nitro–L– arginine methyl esther), SOD 1–3 (superoxide dismutase 1–3), GAPDH (glyceraldehyde-3-phosphate dehydrogenase)

In the case of gene expression of individual SOD isoforms, we noticed major changes in SOD1 expression only in young rats. We observed that 7-NI and L-NAME significantly decreased expression of SOD1 and treatment with 7-NI markedly attenuates this expression compared with L-NAME (Fig. 3a). Different effects of another two SOD isoforms (SOD2 and SOD3) were observed only in adult Wistar rats. Decline of SOD2 expression was recorded after L-NAME, while administration of nNOS inhibitor (7-NI) and eNOS inhibitor (L-NAME) lead to stimulation of expression of the extracellular SOD (SOD3)(Fig. 3b).

Activation of Nrf2 was detected through HO-1 expression. mRNA expression of HO-1 was normalized to “housekeeper” GAPDH. In adult rats we found stimulation of HO-1 gene expression significantly increased only in the L-NAME group (88.4%; L-NAME 1.174 ± 0.13 vs. control 0.623 ± 0.06). 7-NI did not influences HO-1 expression, however a difference in 7-NI and L-NAME group (54.5%; 7-NI 0.76 ± 0.07 vs. L-NAME 1.174 ± 0.13) was observed.

An age-dependent effect on mRNA SOD isoforms has been observed. We found changes among young and adult rats in mRNA SOD2 and SOD3, while SOD1 was not affected (Table 2).

**Fig. 4:**
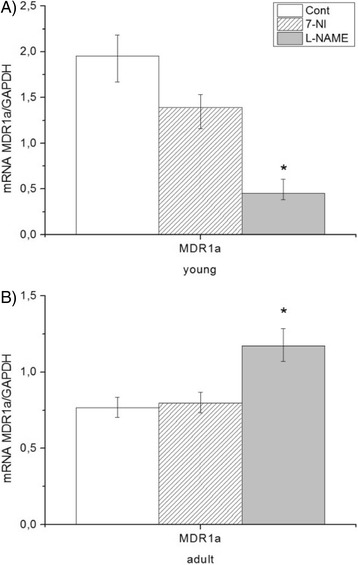
Effect of NOS inhibitors on multidrug resistance of young (**a**) and adult (**b**) Wistar rats. Expression of multidrug resistance MDR1a gene was normalized on expression of GAPDH in the brain stem . Data represent mean ± std.dev.. *P < 0.05, L-NAME vs. control; #*P* < 0.05, 7-NI vs. L-NAME. 7-NI (7-nitroindazole), L-NAME (N^G^–nitro–L– arginine methyl esther), MDR1a (multidrug resistant protein 1a), GAPDH (glyceraldehyde-3-phosphate dehydrogenase)

Gene expression of the detoxification MDR1a genes showed a decrease in the brain stem of young animals (Fig. 4a) and increase in adult rats after chronic administration of L-NAME compared to control and 7-NI group (Fig. 4b).

### Effect of NOS inhibitors on NOS and SOD activity in the brain stem

In young and adult Wistar rats chronic administration of L-NAME significantly decreased the activity of NOS in the brain stem compared with control groups. Treatment with 7-NI does not affect total activity of NOS in young or adult animals (Table 3).

**Table 3:** Effect of NOS inhibitors on NOS and SOD activity in young and adult Wistar rats

Activity	Young rats			Adult rats		
	Control	7-NI	L-NAME	Control	7-NI	L-NAME
NOS (pkat/g proteins)	95,8 ± 76,45	117,4 ± 90,6	-12,1 ± 21,6*^#^	14,9 ± 6,7	16,2 ± 6	1 ± 1*^#^
SOD (U/mg proteins)	1,58 ± 0,5	3,04 ± 0,8*^#^	1,57 ± 0,5	4,96 ± 0,7	5,98 ± 0,8	6,18 ± 1

Activity of SOD were expressed as U/mg of proteins and activity of NOS as pkat/g of proteins. Data represent mean ± std. dev., *P < 0.05, L-NAME vs. control; ^#^ *P* < 0.05, L-NAME vs. 7-NI.7-NI (7-nitroindazole), L-NAME (N^G^-nitro-L-arginine methyl esther), NOS (nitric oxide synthase), SOD (superoxide dismutase)

We observed statistically significant increase of total activity of SOD only after administration of 7-NI in young Wistar rats during measurement of antioxidant response. In young and adult rats, changes in total activity of SOD were not monitored after inhibition of NOS (Tab. 3).

### Fluorescent detection of NO production in cell model with PgP and MRP proteins.

**Fig. 5:**
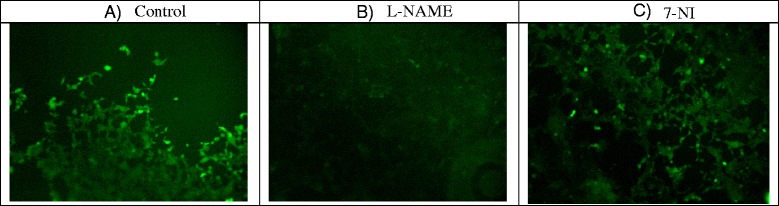
NO production in SH-SY5Y neuronal cell line after NO-inhibition with L-NAME and 7-NI. Data represent mean (intensity density/area) ± std. dev., *P < 0.05, L-NAME, 7-NI vs. control; 7-NI (7-nitroindazole), L-NAME (N^G^-nitro-L-arginine methyl esther)

NO inhibitors in 24 h influenced NO production in SH SY5Y cells containing PgP and MRP proteins. In 7-NI a 8.8% decrease of NO production was observed (control 1.73 ± 0.13 vs 7-NI 1.59 ± 0.16) and in L-NAME, a significant decrease of 34,3% (control 1.73 ± 0.13 vs L-NAME 1.15 ± 0,03, *p < 0.05) (Fig. 5).

## Discussion

### Effect of NOS inhibitors on systolic blood pressure

Many experiments observed that NO-deficiency is tightly related to the development of persistent hypertension. Several NOS inhibitors are used to study NO-deficiency. L-NAME is a widely used NOS blocker, being a non-specific NOS inhibitor. Less frequently, 7-NI is used as a specific blocker of nNOS. Long-term administration of high doses of L-NAME leads to continuous increase in blood pressure and induces structural changes in the cardiovascular system [10]. Hypertrophy of heart and vessel wall is observed after chronic treatment with L-NAME. On the contrary, acute or chronic oral administration of 7-NI does not evoke changes in blood pressure, but hypertrophy of the heart and vessel wall is present [15–18, 20]. We observed different effects of 7-NI and L-NAME on blood pressure. While 7-NI did not alter blood pressure within the 6 weeks of treatment in either age group, L-NAME significantly increased the blood pressure). From these results it seems that unchanged blood pressure after chronic administration of 7-NI should not influence sympathetic outflow, while it seems that L-NAME was mediated by the sympathetic drive [58]. In intraperitoneal and intravenous administration of 7-nitroindazole (7-NI) was also observed that it does not affect mean arterial blood pressure in mice and rats [66]. In the studies with combined administration of 7-nitroindazole and L-NAME comparing to their individual administrated to Wistar rats had been observed different effect on blood pressure. Combinations of L-NAME and 7-NI lead to decrease of blood pressure in hypertension animals comparing to L-NAME treated hypertensive animals [67]. It was also shown, that effect of individual 7- NI administration performed blood pressure-independent hypotrophy of the heart, kidneys and conduit arteries [20]. An alternative explanation of the lack of effect of 7-nitroindazole on blood pressure are cardiac and vascular structural changes in Wistar rats [20, 67]. Increases in BP are usually accompanied by cardiac hypertrophy. While cardiac hypertrophy was repeatedly observed after L-NAME administration to Wistar rats, 7-NI evoked an opposite (hypotrofic) effect on the hearts of Wistar rats [18, 68–70]. Cardiac hypotrophy induced by 7-NI may participate in eventual heart failure and myocardial structural changes would have to impact on the unchanged peripheral resistance in Wistar rats [67].

In young rats treated with L-NAME we observed blood pressure to increase up to 140 mmHg, which represents borderline hypertention (20.7% increase of blood pressure),while adult rats achieved the level of high blood pressure characteristic for hypertensive animals (166 mmHg, 54.3% increase of blood pressure) (Tab. 1). These changes in blood pressure were associated with differences in other measured parameters (NO, AT1R pathway, antioxidant and detoxicant responses) in young and adult rats.

### Effect of NOS inhibitors on expression and activity of NOS in brain stem

7-nitroindazole is used as a specific blocker of neuronal NOS at certain concentrations [71]. In our young Wistar rats we observed a decrease in nNOS mRNA in the 7-NI group (Fig. 1a), while in adult rats 7-NI attenuated only the expression of eNOS (Fig. 1b). NOS activity was not influenced (Tab.3). L-NAME treatment had the same effect on nNOS (in young animals) (Fig. 1a) and eNOS (in adult animals) as 7-NI, but the opposite effect on nNOS in adult rats (Fig. 1b). In the case of L-NAME treatment, NOS activity was decreased in young and adult rats (Tab. 3). Arginine from one side and L-NAME (exogenous) or ADMA (endogenous) are competitive substrates for activation and blockade of NOS activity, while 7-NI did not compete with arginine, so the final effect on total NOS activity can be different. In other studies acute and/or chronic administration of L-NAME showed inhibition of NOS activity in the brain stem [72–74]. However, L-NAME has unequal influence on expression of NOS isoforms depending on the duration of treatment and brain areas. Expression of eNOS proteins after a 4-week administration of L-NAME was unchanged in the brain stem. Decline of eNOS was recorded after a 7 weeks treatment [75]. Administration of L-NAME suppresses expression of endothelial and neuronal NOS in hippocampus of rats [76].

### Effect of NOS inhibitors on AT1-NAD(P)H oxidase pathway in brain stem

Several studies have shown that NOS inhibition stimulates the presence of oxidative stress. The administration of L-NAME is often associated with increased production of secondary products of oxidative stress [77–80]. A short-term (2 weeks) administration of L-NAME reduces the activity of glutathione peroxidase and stimulates lipid peroxidation and the level of TBARS in rat brain [81]. Chronic administration of L-NAME results in an increase of malondialdehyde (MDA) in the hippocampal region of the brain [82]. The administration of L-NAME increases production of superoxide anion in vessels and production of MDA in the liver [83, 84], and stimulates production of plasma renin [85] and AngII. Short term and chronic inhibition with L-NAME leads to an increase of plasma angiotensin II [86]. Similarly, Maneesai et al. (2016) observed upregulation of AT1 receptors and increased plasma angiotensin II after chronic inhibition with L-NAME [87]. In our chronic studies we found stimulation of the AT1R-NAD(P)H oxidase pathway in adult rats (Fig. 2b). The membrane-bound p22phox subunits are essential in maintaining a stable unit capable of supporting electron transfer for superoxide generation [88]. The p22phox subunit is common to all NADPH oxidase isoforms (and therefore associates directly or indirectly with all known NADPH oxidase subunits) [89]. Fukui et al. (1997) observed that NADPH oxidase-specific production of superoxide is increased during Ang II-induced hypertension. Activation of the NADPH oxidase system was accompanied by upregulation of mRNA levels of one or several components of this oxidase system, including the p22phox in rat aorta [90]. Correlation between NOS and mRNA AT1 receptors was found in brain, but is not in correlation with the presence of AT2 mRNA receptors [91]. L-NAME has different impact on expression of AT1R in different brain areas, because it stimulates expression of AT1R in the hypocampus, but not in the cortex after a 4 week administration [92]. L-NAME affected these changes, while 7-NI did not. Several studies have confirmed that nNOS blockers, like 7-NI, completely inhibit the secretion of renin, a key enzyme in production of Ang II [15, 93]. These processes reduce the effect of the renin-angiotensin system [94], which affects the activity of NAD(P)H oxidase and production of ROS. We did not observe any changes in AT1R- NAD(P)H oxidase pathway in the 7-NI group, which is Ang II dependent.

Our results show that stimulation of the radical signaling pathway through AT1R-NAD(P)H-oxidase is different for various age groups and NOS inhibitors. This radical pathway in the brain stem is not significantly influenced in young rats (Fig. 2a), while it is stimulated in adult rats (Fig. 2b).

#### Antioxidant and detoxification responses: antioxidant response, Nrf2 activation and multidrug resistance in the brain stem

NOS inhibition affects oxidative stress in the brain and also induces changes in the antioxidant response. Previous studies showed changes in the total plasma activity of superoxide dismutase after L-NAME treatment [83, 95]. Chronic L-NAME-induced inhibition of NOS attenuates activity of SOD and glutathion peroxidase, but stimulates catalase activity in kidneys [96, 97]. Cardoso et al. [97] observed that SOD and catalase activities decreased in L-NAME induced hypertension. In short-term administration, Oktar et al. (2010) observed that N_G_-nitro-L-arginine (L-NNA) stimulated SOD activity, but not catalase activity in brain [98]. These differences may depend on dose, age and/or duration of the substance administration. In addition, NOS inhibition causes tissue-dependent changes in levels of antioxidants. In the present study we found different age-dependent responses among NOS inhibitors. In the L-NAME group, there were no changes in SOD activities in young and adult rats, while in the 7-NI group, SOD activity was significantly increased only in the young Wistar rats (Tab. 3). This increase was caused by a pathway different than through AT1R-NAD(P)H oxidase, because no changes in the expression of AT1R and p22phox were seen in this group (Fig. 3a). Many studies have shown a protective effect of 7-NI against induced neurotoxicity and experimental stroke [99–102], where overexpression and deficiency in SOD mice models of neurotoxicity plays an important role [103, 104]. It was also observed that 7-NI inhibited monoamine oxidase A in brain and acted as a potent antioxidant [105].We observed that the AT1R and p22phox pathways have not been changed in the 7-NI group (Fig. 2a) and expression of the SOD1 isoform was diminished (Fig. 3a), while SOD activity was increased (Tab. 3). The main function of SOD3 is dismutation of superoxide anions generated in the extracellular medium by biochemical reactions involving membrane-bound enzymes such as xanthine oxidase and NAD(P)H oxidase [106]. In normal conditions, SOD3 was found to minimize O_2_^∸^ levels, protecting endogenously produced NO at a sufficient level to maintain cerebral vascular tone and reactivity. SOD3 was found to increase the vasodilatory effect of endogenously produced NO in the brain [107]. Stimulation of this SOD3 isoform was observed only in adult Wistar rats in both NOS inhibitor groups (Fig. 3b), where we also found an increase in AT2R expression. Several other studies showed that AT2R-stimulation attenuates hypertensive end-organ damage in kidneys, vasculature and the brain [36, 38, 39].

Cytoprotective responses in brain were observed through the Nrf2 activation pathway (detoxification phase II) and/or MDR1 pathway (detoxification phase III). The Nrf2/ Keap1/ARE signaling pathway is an important regulator of cellular oxidants and electrophile stress through induction of antioxidant and detoxification genes such as SOD3 and HO-1 [48]. Activation of Nrf2 is often detected through HO-1 expression. We found that stimulation of HO-1 gene expression was observed only in adult rats after L-NAME treatment. Induction of HO-1 expression by Nrf2 has hypotensive effects and is upregulated in spontaneously hypertensive rats, which suggests its role in hypertension [49, 108].

The P-gp efflux transporter is a mechanism involved in the protection of CNS against exogenous drugs. P-gp is coded by *MDR1a* and *MDR1b* genes in rodent brain, but only *MDR1a* is localized in brain capillaries. This efflux transporter mediates the export of drugs from the blood-brain barrier (BBB), where it prevents many drugs from entering into the CNS [50, 53]. We observed decreased expression of *MDR1a* genes in the brain stem of young animals (Fig. 4a) and increased expression in adult rats after chronic administration of L-NAME (Fig. 4b), which should correlate with P-glycoprotein activity in the BBB.Wang et al. [109] have shown that Nrf2 activation with sulforaphane in vivo or in vitro also increases the expression and transport activity of ATP-driven drug efflux pumps at the blood–brain barrier (P-gp) [105]. We also observed positive correlation between Nrf2 and MDR1a in adult rats after chronic L-NAME treatment.

Oral way of treatment with NOS inhibitors (L-NAME and 7-NI) used in our study is a commonly used method for studying NO-deficient hypertension. Direct action of the pharmacological agents on brain were expected based on chemical ADMET (Absorption, Distribution, Metabolism, Excretion and Toxicity profiles) properties of 7-nitroindazole and N(G)-Nitro-L-arginine methyl esther. The ADMET structure-activity relationship server (admetSAR) is a comprehensive knowledge base and a tool for predicting ADMET properties of drug candidates [110]. ADMET Predicted Profile modeled by admetSAR showed that 7-nitroindazole has high probability (0.9745) of blood brain barrier (BBB+) absorption and high probability (0.9911) of intestinal absorption (IA+). L-NAME has probability of blood brain barrier absorption 0.7210 and probability of intestinal absorption of 0.6725. Both inhibitors are not substrate for P-glycoprotein (probability: 7-NI – 0.8679, L-NAME – 0.5511). Transport mechanisms of L-NAME are not identified, but amino acid transporters are present in the blood brain barrier. L-NAME is an analog of L-arginine, which is transported through the cationic amino acids transporter system y+. These transporters may be one of the possible mechanism of L-NAME transportation through blood brain barrier [111].

### NO- detection and model of blood brain barrier in SH-SY5Y cells

The effect of orally administered NO inhibitors in brain stem critically depends on their ability to cross the blood brain barrier (BBB). The BBB protects the brain from potentially harmful substances [50] and is formed by the brain microvascular endothelial cells (BMVEC), pericytes and astrocytes [112]. BMVEC cells contain active drug efflux transporters: the ABC efflux transporter, P-glycoprotein that actively transports lipophilic drugs, and members of the multidrug resistance protein family [50].

The ADMET database (http://www.simulations-plus.com/software/admet-property-prediction-qsar/) provides predictions of BBB crossing probabilities for drugs and other substances. For L-NAME and 7-NI, it predicts BBB crossing probabilities of 72.1% and 97.45%, respectively.

To obtain a direct estimate of the ability of L-NAME and 7-NI to act across the BBB, we used the SH-SY5Y neuroblastoma cell line. Cells of this line endogenously express the major BBB transporters [113], notably the P-glycoprotein (Pgp) and MRP proteins, and we can therefore expect the interior of the cells to be under protection similar to the BBB.

We studied the influence of L-NAME and 7-NI on NO production in the SH-SY5Y cells. In a small study we found a significant decrease in NO production in the presence of L-NAME, and a visible, yet statistically nonsignificant decrease in the presence of 7-NI.

To further complicate the matter, we observed differences in MDR1a expression in our animal chronic administration study between young and adult rats. The presence of efflux proteins in the SH-SY5Y cell line and their effect on NO production should be studied in more details in a further, larger study.

## Conclusion

Our results show that chronic inhibition of NOS by two different NOS inhibitors (7-NI, L-NAME) has age-dependent effect on radical signaling (AT1R-NAD(P)H oxidase pathway) and antioxidant (Nrf2 activation) and detoxification (MDR1a transporters) response in Wistar rats. In young rats, major effect wasobserved in antioxidant response (SOD activity) and expression of AT2R induced by 7-NI. This stimulation of antioxidant system suggests aneuroprotective effect of 7-NI in brain stem of young Wistar rats. In adult rats, treatment with L-NAME led to activation of AT1R-NAD(P)H oxidase pathway following stimulation SOD3 expression as a protection of NO in brain stem, AT2R expression as a protection against brain damage;other lines of the defense system were activated as well Nrf2-ARE pathway and efflux transporter. Our data suggest that changes of radical signaling, antioxidant and detoxification response can be important in the development of hypertension.

## Additional file

Additional file 1: Data outputs_statistics. (PDF 278 kb)

## Abbreviations

7-NI: 7-nitroindazole
AngII: Angiotensin II
ARE: Antioxidant response element
AT1R: Angiotensin receptor 1
AT2R: Angiotensin receptor 2
BBB: Blood-brain barrier
CNS: Central nervous system
GAPDH: Glyceraldehyde-3-phosphate dehydrogenase
H_2_O_2_: Hydrogen peroxide
HO-1: Heme oxygenase-1
iNOS: Inducible NOS (NOS2)
Keap1: Kelch-like ECH-associated protein 1
L-NAME: N^G^-nitro-L-arginine methyl esther
L-NNA: N^G^-nitro-L-arginine
MDR1: Multidrug resistance gene (MDR1a, MDR1b)
mRNA: Messenger RNA
nNOS: Neuronal NOS (NOS1)
NO: Nitric oxide
NOS: Endothelial NOS (NOS3)
Nox: NAD(P)H oxidase
Nrf2: Nuclear factor-E2-related factor
NTS: Nucleus tractus solitarii
O_2_∸: Superoxide
p22phox: Subunit of NAD(P)H oxidase
P-gp: P-glycoprotein
ROS: Reactive oxygen species
RVLM: Rostral ventrolateral medulla
SNS: Sympathetic nervous system
SOD: Superoxide dismutase
SOD1: Copper-zinc SOD (Cu/ZnSOD)
SOD2: Manganese SOD (MnSOD)
SOD3: Extracellular SOD (ecSOD)
