# Supplementary material for: Age-dependent redox status in the brain stem of NO-deficient hypertensive rats
Source: J Biomed Sci. 2017 Sep 11;24:72. doi: 10.1186/s12929-017-0366-4 (PMC5594586; doi:10.1186/s12929-017-0366-4)
Supplement: Additional file 1: — Data outputs_statistics. (PDF 278 kb) [file 12929_2017_366_MOESM1_ESM.pdf]

Data analysis for a group of measurements

This is analysis script for data that use Reference 2 ("GAPDH2").

The analysis script does the following: 1. Load and re-organize the data. 2. Record calibration curves $C \sim \log(Cycle)$ for all measurement, including reference. 3. Fit linear model $Cycle \sim Gene : Group + Location$, use the getOutliers function from the extremevalues R package to detect outliers. Remove outliers from the fit, refit and replace outliers with model predictions. Use gene calibration curves to re-map corrected cycles to concentrations. 4. Create LogRat = $\log(C/C_{ref})$ as response variable. 5. Do some exploratory plotting 6. Fit a linear model LogRat ~ Gene * Group + Location to the data. 7. Detect outliers again using the getOutliers function and replace them with linear fit predictions. 5. Re-fit the model. 6. Calculate contrasts (7-NI - KONTROLA, L-NAME - KONTROLA, L-NAME - 7-NI) for each gene, and calculate their significance using 3 methods: a. Single-step Bonferoni adjustment using function glht from R-package multcomp b. Westfall-Young resampling-based free step-down method c. Benjamini-Yekutieli adjustment of p-values 7. Compare to see if p-values are consistent 8. Make plots of data and results

Load data

Load and merge the csv's containing gene expression data. The data are directly taken from Excel. Data marked as outliers are excluded.

```
##    Location    Group Cycle         C          Gene
## 1        4E KONTROLA 24.61  25.15787 GAPDH_FOR_ENOS
## 2        4E KONTROLA 24.38  31.02474 GAPDH_FOR_ENOS
## 3        8E KONTROLA 27.72   1.44032 GAPDH_FOR_ENOS
## 4        8E KONTROLA 27.28   2.15049 GAPDH_FOR_ENOS
## 5        4F KONTROLA 25.18  14.91895 GAPDH_FOR_ENOS
## 6        4F KONTROLA 25.04  16.90031 GAPDH_FOR_ENOS
## 7        8F KONTROLA 36.46        NA GAPDH_FOR_ENOS
## 8        8F KONTROLA 28.27   0.86456 GAPDH_FOR_ENOS
## 9        4G KONTROLA 25.25  13.97613 GAPDH_FOR_ENOS
## 10       4G KONTROLA 25.65   9.64860 GAPDH_FOR_ENOS
## 11       8G KONTROLA 24.32  32.97945 GAPDH_FOR_ENOS
## 12       8G KONTROLA 24.44  29.44046 GAPDH_FOR_ENOS
## 13      12E     7-NI 26.93   2.97573 GAPDH_FOR_ENOS
## 14      12E     7-NI 26.54   4.24731 GAPDH_FOR_ENOS
## 15      16E     7-NI 25.88   7.81642 GAPDH_FOR_ENOS
## 16      16E     7-NI 25.84   8.11699 GAPDH_FOR_ENOS
## 17      12F     7-NI 25.89   7.75030 GAPDH_FOR_ENOS
## 18      12F     7-NI 25.84   8.12077 GAPDH_FOR_ENOS
## 19      16F     7-NI 24.37  31.50016 GAPDH_FOR_ENOS
## 20      16F     7-NI 24.35  31.90786 GAPDH_FOR_ENOS
## 21      12G     7-NI 23.16  95.96038 GAPDH_FOR_ENOS
## 22      12G     7-NI 23.72  56.96787 GAPDH_FOR_ENOS
## 23      16G     7-NI 23.59  64.55822 GAPDH_FOR_ENOS
## 24      16G     7-NI 23.68  59.14144 GAPDH_FOR_ENOS
## 25      20E   L-NAME 24.03        NA GAPDH_FOR_ENOS
## 26      20E   L-NAME 24.50  27.87211 GAPDH_FOR_ENOS
## 27      20F   L-NAME 24.11  40.00418 GAPDH_FOR_ENOS
## 28      20F   L-NAME 24.23  35.82644 GAPDH_FOR_ENOS
## 29      24F   L-NAME 24.52  27.25456 GAPDH_FOR_ENOS
## 30      24F   L-NAME 24.87  19.72896 GAPDH_FOR_ENOS
## 31      20G   L-NAME 22.48 179.65227 GAPDH_FOR_ENOS
## 32      20G   L-NAME 22.40 192.42992 GAPDH_FOR_ENOS
```

```
## 33      24G   L-NAME 24.51  27.62451 GAPDH_FOR_ENOS
## 34      24G   L-NAME 24.68  23.68868 GAPDH_FOR_ENOS
```

```
## Loading required package: ggplot2
```

```
## Warning: Removed 2 rows containing missing values (geom_point).
```

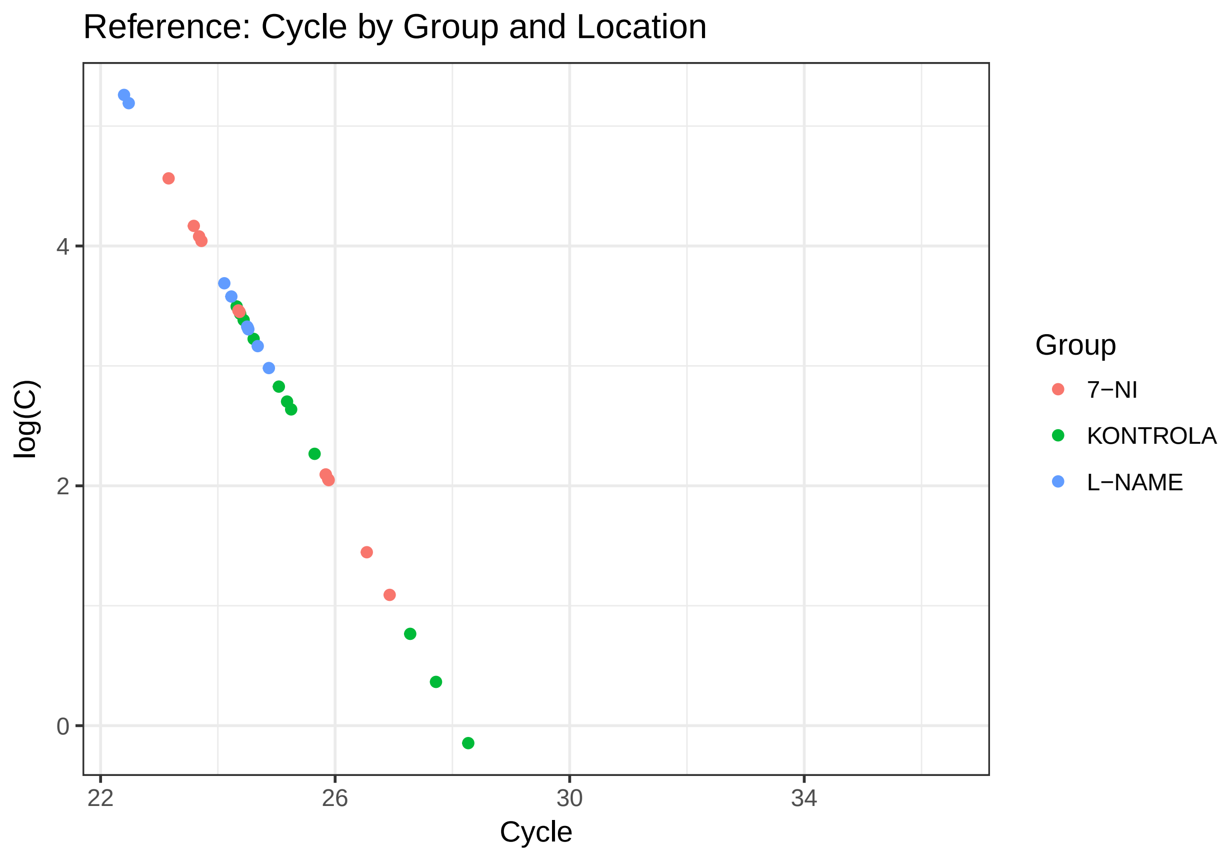


```
##
## Call:
## lm(formula = log(C) ~ Cycle, data = reference)
##
## Residuals:
##        Min         1Q     Median         3Q        Max
## -0.0045254 -0.0019477  0.0003691  0.0020911  0.0033850
##
## Coefficients:
##               Estimate Std. Error t value Pr(>|t|)
## (Intercept) 25.8930980  0.0076638    3379   <2e-16 ***
## Cycle       -0.9210489  0.0003068   -3002   <2e-16 ***
## ---
## Signif. codes:  0 '***' 0.001 '**' 0.01 '*' 0.05 '.' 0.1 ' ' 1
##
## Residual standard error: 0.002357 on 30 degrees of freedom
##   (2 observations deleted due to missingness)
## Multiple R-squared:      1,  Adjusted R-squared:      1
## F-statistic: 9.014e+06 on 1 and 30 DF,  p-value: < 2.2e-16
```

```
## Warning: Removed 2 rows containing missing values (geom_point).
```

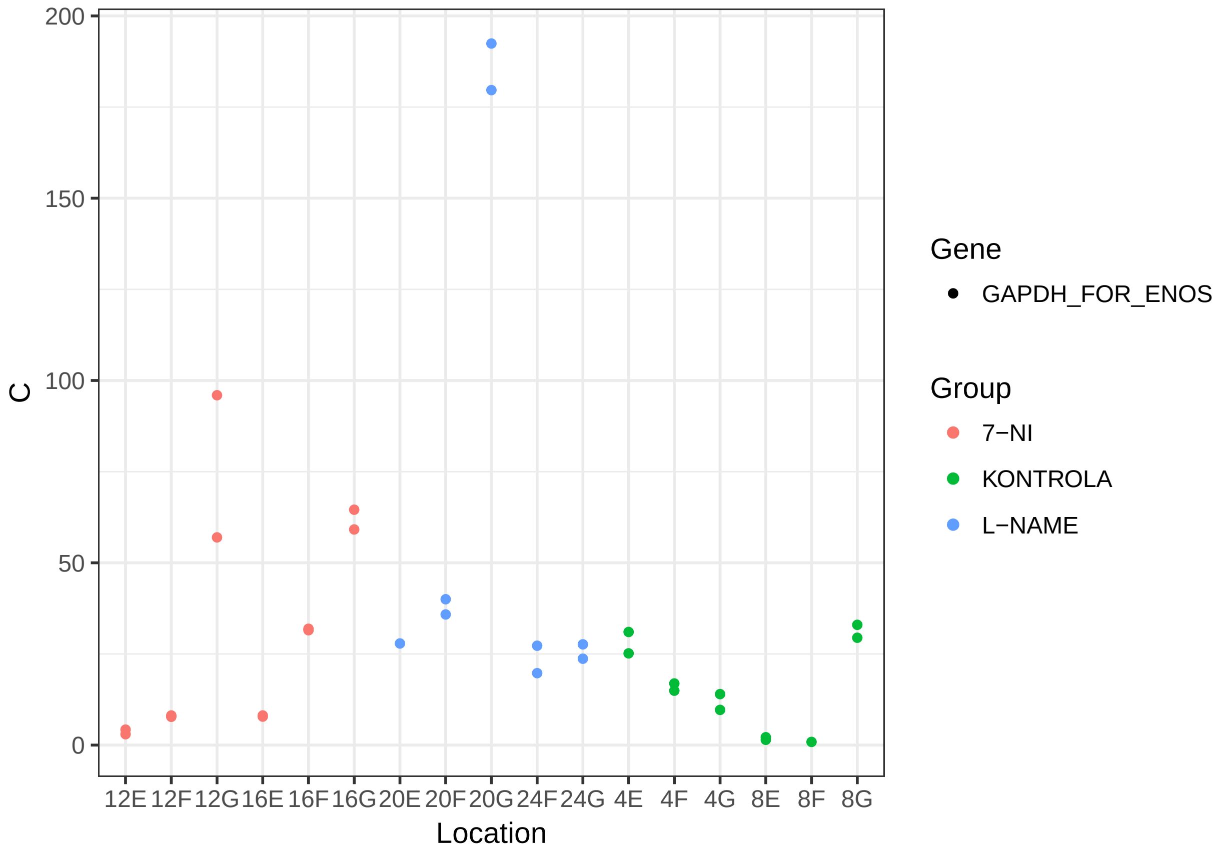


```
##    Location    Group Cycle         C Gene      Cref       LogRat
## 1        4E KONTROLA 25.13 23.40655 eNOS  25.15787 -0.07215487
## 2        4E KONTROLA 25.31 20.62494 eNOS  31.02474 -0.40828393
## 3        8E KONTROLA 27.87  3.53099 eNOS   1.44032  0.89671297
## 4        8E KONTROLA 27.93  3.38676 eNOS   2.15049  0.45417799
## 5        4F KONTROLA 26.07 12.21931 eNOS  14.91895 -0.19961473
## 6        4F KONTROLA 26.18 11.35273 eNOS  16.90031 -0.39787372
## 7        8F KONTROLA 30.21  0.70028 eNOS        NA          NA
## 8        8F KONTROLA 29.92  0.85420 eNOS   0.86456 -0.01205535
## 9        4G KONTROLA 26.85  7.13067 eNOS  13.97613 -0.67294568
## 10       4G KONTROLA 27.18  5.68889 eNOS   9.64860 -0.52829768
## 11       8G KONTROLA 25.66 16.20418 eNOS  32.97945 -0.71061541
## 12       8G KONTROLA 25.69 15.89199 eNOS  29.44046 -0.61655471
## 13      12E     7-NI 28.24  2.73060 eNOS   2.97573 -0.08596802
## 14      12E     7-NI 28.23  2.74681 eNOS   4.24731 -0.43584560
## 15      16E     7-NI 27.44  4.76634 eNOS   7.81642 -0.49464793
## 16      16E     7-NI 27.44  4.74354 eNOS   8.11699 -0.53717570
## 17      12F     7-NI 27.23  5.50878 eNOS   7.75030 -0.34138837
## 18      12F     7-NI 27.20  5.60288 eNOS   8.12077 -0.37114423
## 19      16F     7-NI 25.89 13.81611 eNOS  31.50016 -0.82415732
## 20      16F     7-NI 26.07 12.26612 eNOS  31.90786 -0.95601138
## 21      12G     7-NI 26.04 12.50009 eNOS  95.96038 -2.03819955
## 22      12G     7-NI 27.26       NA eNOS  56.96787          NA
## 23      16G     7-NI 25.81 14.61225 eNOS  64.55822 -1.48570723
## 24      16G     7-NI 25.92 13.55469 eNOS  59.14144 -1.47319925
## 25      20E   L-NAME 26.08 12.15571 eNOS        NA          NA
```

```
## 26      20E   L-NAME 28.38       NA eNOS  27.87211          NA
## 27      20F   L-NAME 25.61 16.81545 eNOS  40.00418 -0.86668584
## 28      20F   L-NAME 26.02 12.64337 eNOS  35.82644 -1.04155320
## 29      24F   L-NAME 26.30 10.45606 eNOS  27.25456 -0.95803913
## 30      24F   L-NAME 26.55  8.75525 eNOS  19.72896 -0.81243409
## 31      20G   L-NAME 23.15 92.21840 eNOS 179.65227 -0.66686347
## 32      20G   L-NAME 23.82 57.91399 eNOS 192.42992 -1.20077306
## 33      24G   L-NAME 26.72  7.82111 eNOS  27.62451 -1.26187693
## 34      24G   L-NAME 27.24  5.47251 eNOS  23.68868 -1.46525992

## [1] "Number of data rows: 34"

##    Location    Group Cycle        C Gene     Cref      LogRat
## 1        4E KONTROLA 25.13 23.40655 eNOS 25.15787 -0.07215487
## 3        8E KONTROLA 27.87  3.53099 eNOS  1.44032  0.89671297
## 4        8E KONTROLA 27.93  3.38676 eNOS  2.15049  0.45417799
## 5        4F KONTROLA 26.07 12.21931 eNOS 14.91895 -0.19961473
## 6        4F KONTROLA 26.18 11.35273 eNOS 16.90031 -0.39787372
## 8        8F KONTROLA 29.92  0.85420 eNOS  0.86456 -0.01205535
## 9        4G KONTROLA 26.85  7.13067 eNOS 13.97613 -0.67294568
## 10       4G KONTROLA 27.18  5.68889 eNOS  9.64860 -0.52829768
## 12       8G KONTROLA 25.69 15.89199 eNOS 29.44046 -0.61655471
## 13      12E     7-NI 28.24  2.73060 eNOS  2.97573 -0.08596802
## 14      12E     7-NI 28.23  2.74681 eNOS  4.24731 -0.43584560
## 15      16E     7-NI 27.44  4.76634 eNOS  7.81642 -0.49464793
## 16      16E     7-NI 27.44  4.74354 eNOS  8.11699 -0.53717570
## 17      12F     7-NI 27.23  5.50878 eNOS  7.75030 -0.34138837
## 18      12F     7-NI 27.20  5.60288 eNOS  8.12077 -0.37114423
## 29      24F   L-NAME 26.30 10.45606 eNOS 27.25456 -0.95803913
## 30      24F   L-NAME 26.55  8.75525 eNOS 19.72896 -0.81243409
## 33      24G   L-NAME 26.72  7.82111 eNOS 27.62451 -1.26187693
## 34      24G   L-NAME 27.24  5.47251 eNOS 23.68868 -1.46525992

## [1] "Before:"

## [1] "7-NI"     "KONTROLA" "L-NAME"

## [1] "After:"

## [1] "KONTROLA" "7-NI"     "L-NAME"

## [1] "Before:"

##  [1] "12E" "12F" "16E" "24F" "24G" "4E"  "4F"  "4G"  "8E"  "8F"  "8G"

## [1] "After:"

##  [1] "12E" "12F" "16E" "24F" "24G" "4E"  "4F"  "4G"  "8E"  "8F"  "8G"
```

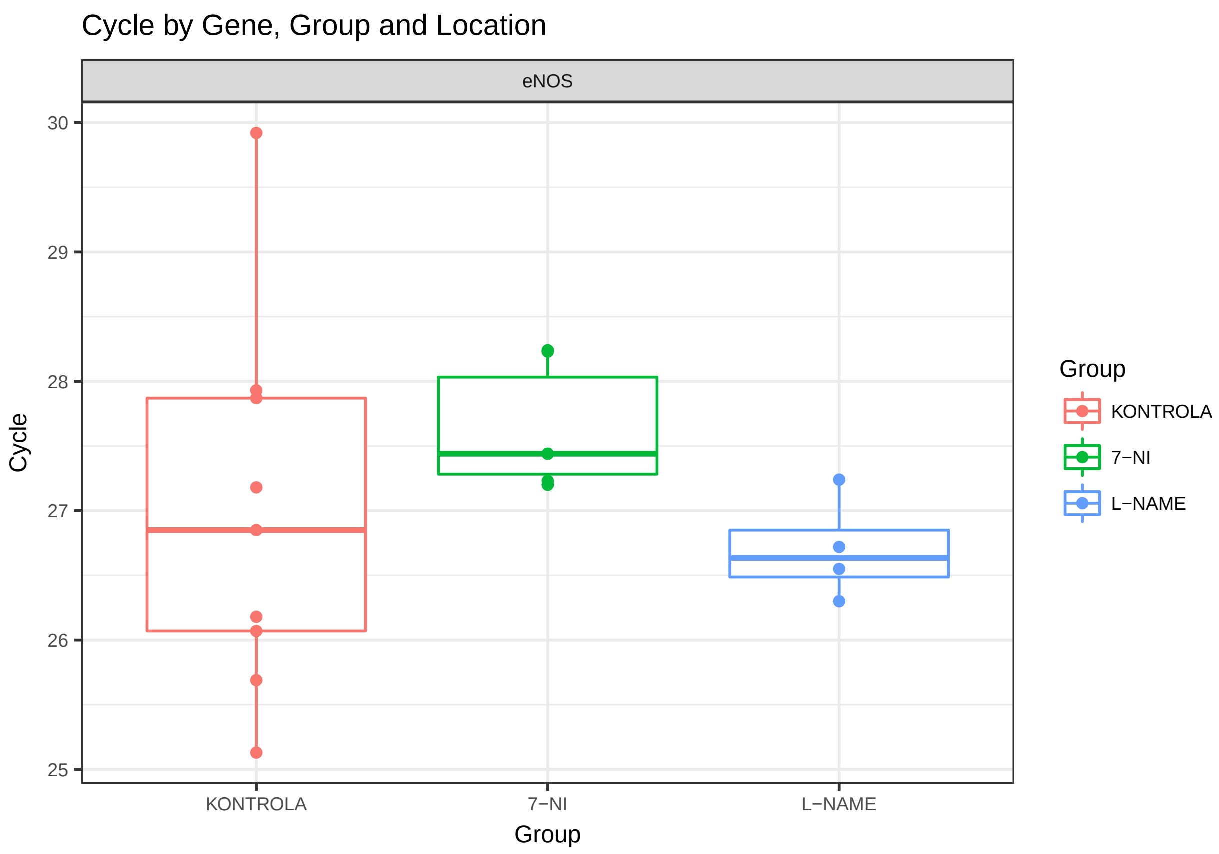

Cycle by Gene, Group and Location
eNOS
30
29
28
27
26
25
Cycle
KONTROLA
7−NI
L−NAME
Group
Group
KONTROLA
7−NI
L−NAME

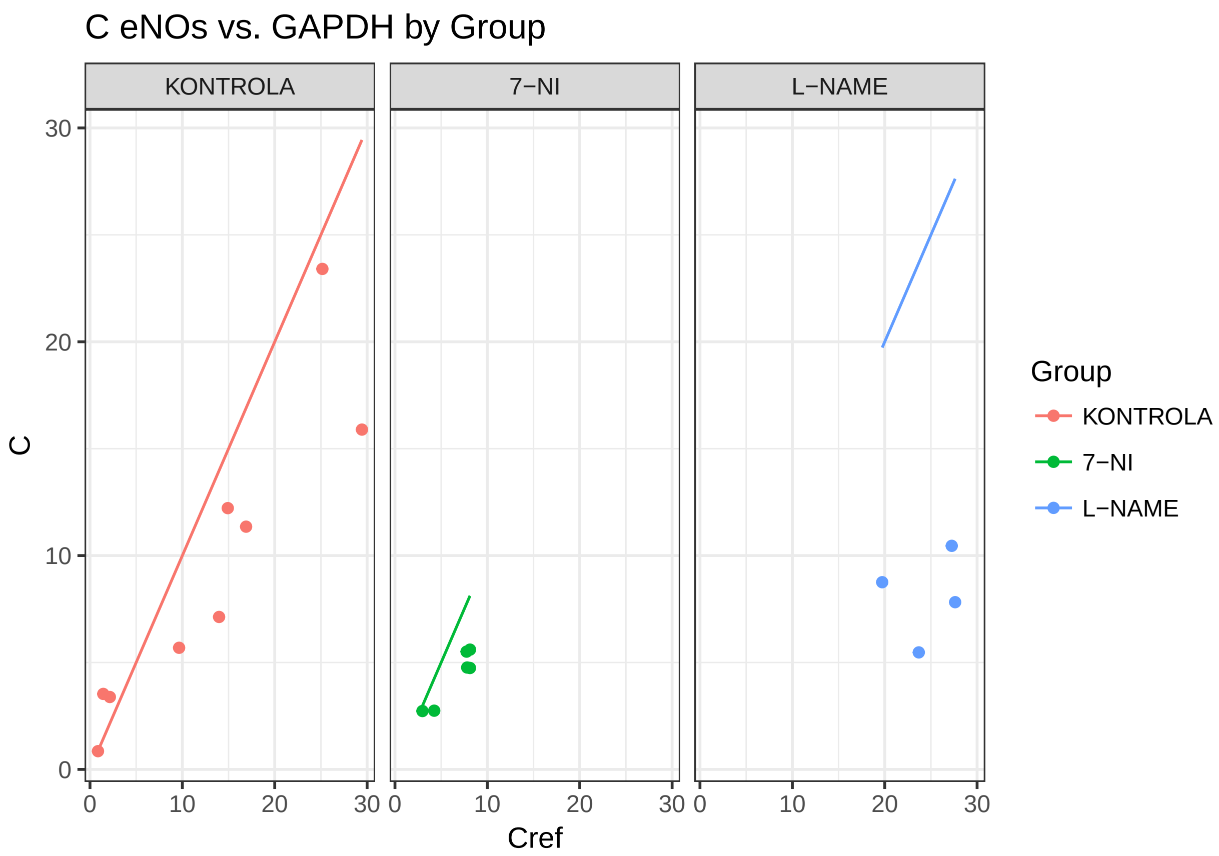

C eNOs vs. GAPDH by Group
KONTROLA
7−NI
L−NAME
30
20
10
0
0
10
20
30
C
Cref
Group
KONTROLA
7−NI
L−NAME

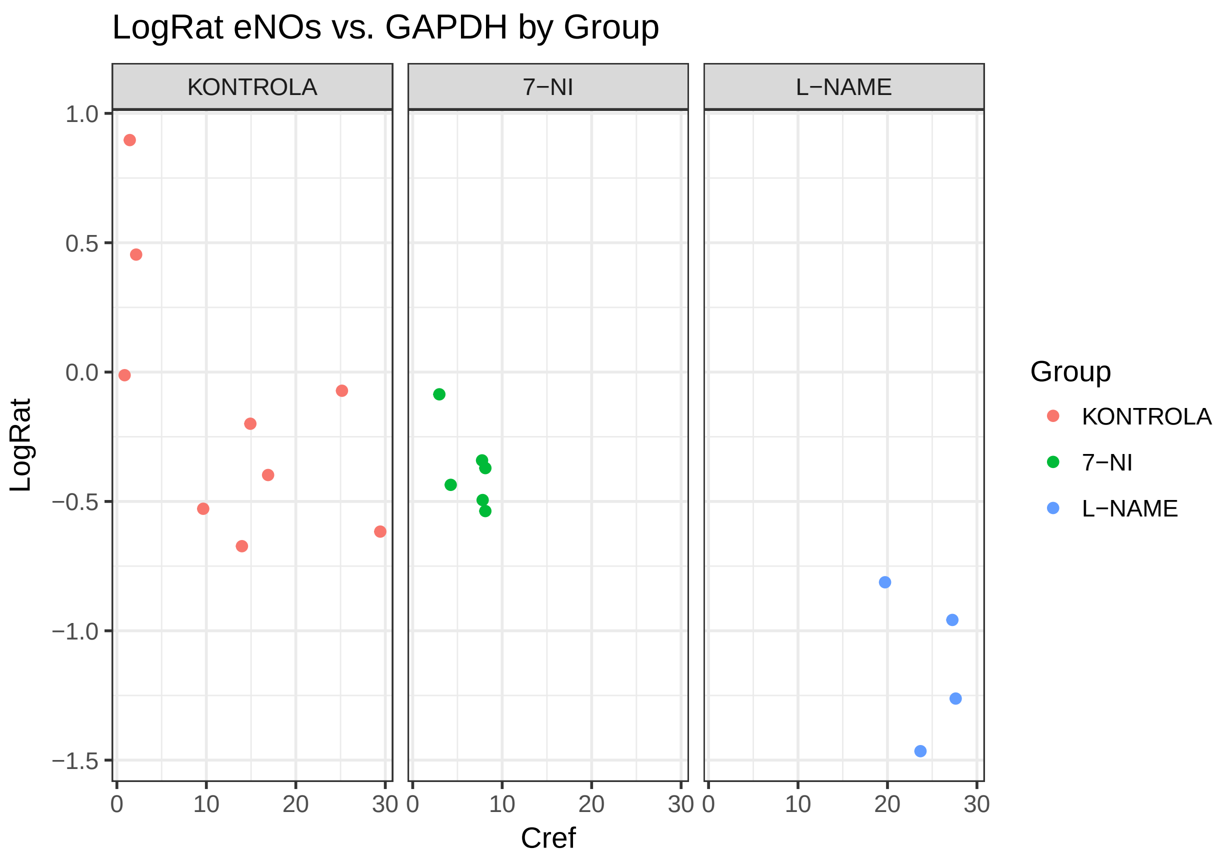

LogRat eNOs vs. GAPDH by Group
KONTROLA
7−NI
L−NAME
1.0
0.5
0.0
−0.5
−1.0
−1.5
LogRat
0
10
20
30
Cref
Group
KONTROLA
7−NI
L−NAME

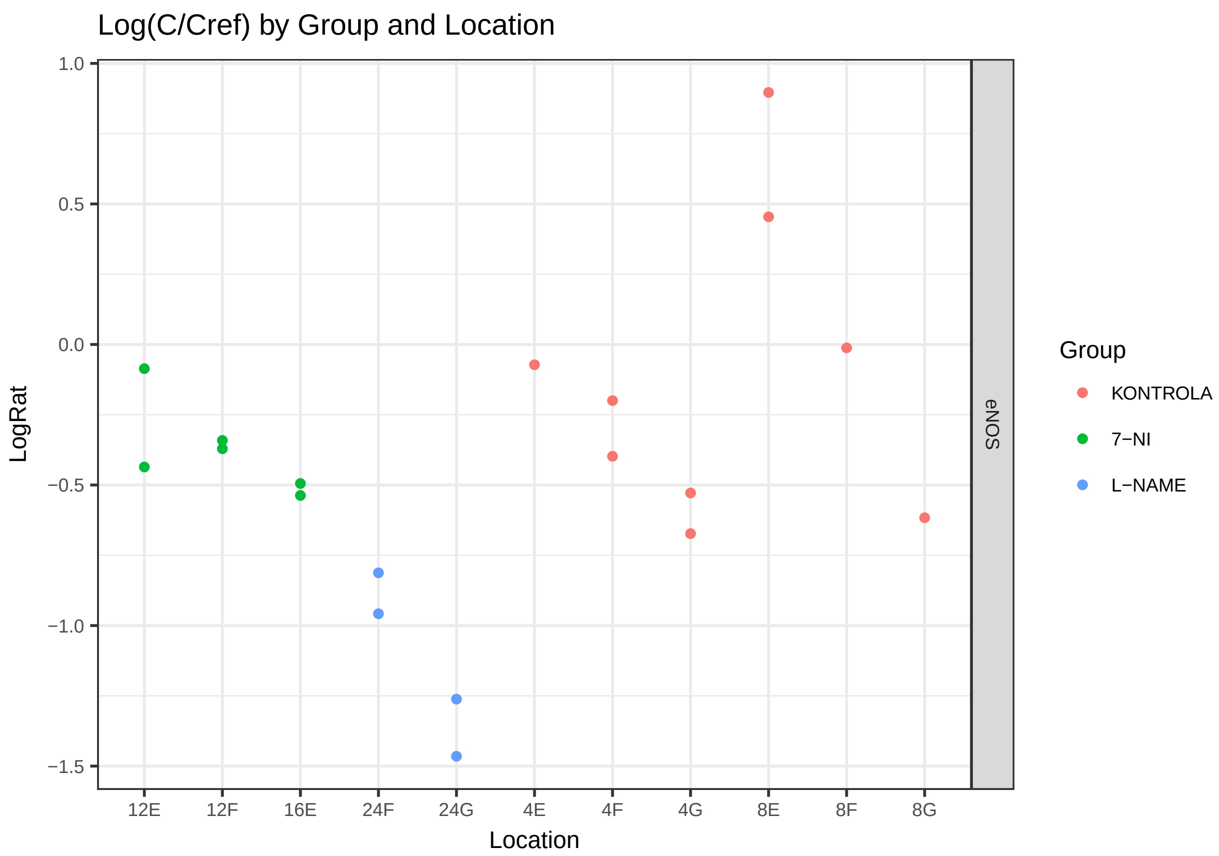

Fit linear model to data and detect outliers.

The model says: Express each log(C/Cref) value as $\mu_0 + \mu_{Group}$

```
##
## Call:
## lm(formula = LogRat ~ Group, data = measurement)
##
## Residuals:
##      Min       1Q   Median       3Q      Max
## -0.54532 -0.21487 -0.05815  0.14097  1.02434
##
## Coefficients:
##             Estimate Std. Error t value Pr(>|t|)
## (Intercept)  -0.1276     0.1336  -0.956 0.353490
## Group7-NI    -0.2501     0.2112  -1.184 0.253609
## GroupL-NAME  -0.9968     0.2408  -4.140 0.000769 ***
## ---
## Signif. codes:  0 '***' 0.001 '**' 0.01 '*' 0.05 '.' 0.1 ' ' 1
##
## Residual standard error: 0.4007 on 16 degrees of freedom
## Multiple R-squared:  0.5184, Adjusted R-squared:  0.4582
## F-statistic: 8.611 on 2 and 16 DF,  p-value: 0.002894
```

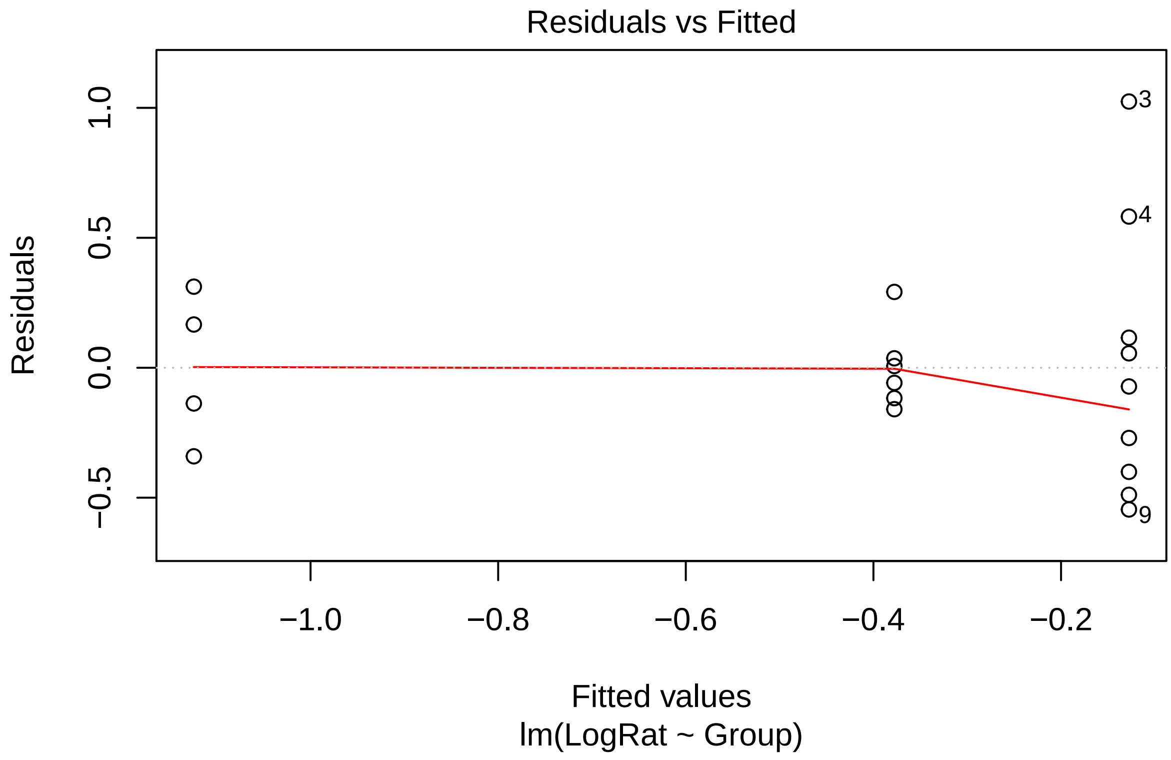

Residuals vs Fitted
Residuals
1.0
0.5
0.0
−0.5
3
4
9
−1.0
−0.8
−0.6
−0.4
−0.2
Fitted values
lm(LogRat ~ Group)

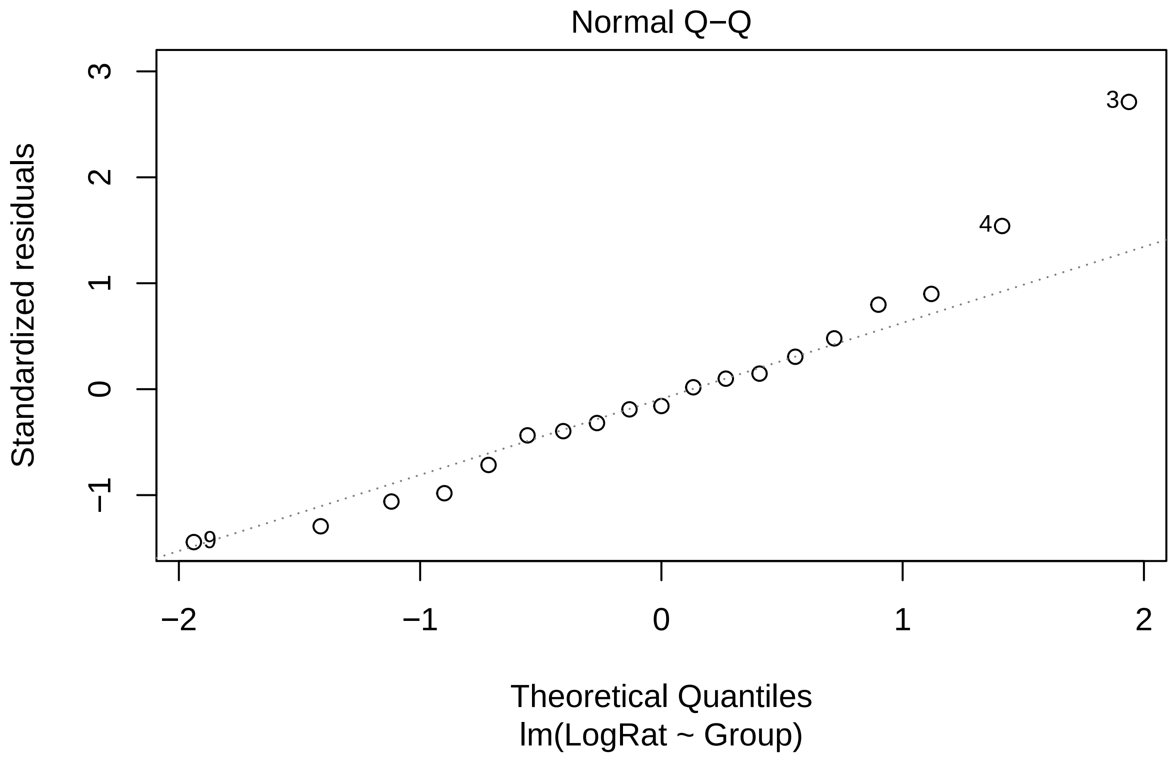
Normal Q−Q
Standardized residuals
Theoretical Quantiles
lm(LogRat ~ Group)
−2
−1
0
1
2
3
9
4
3

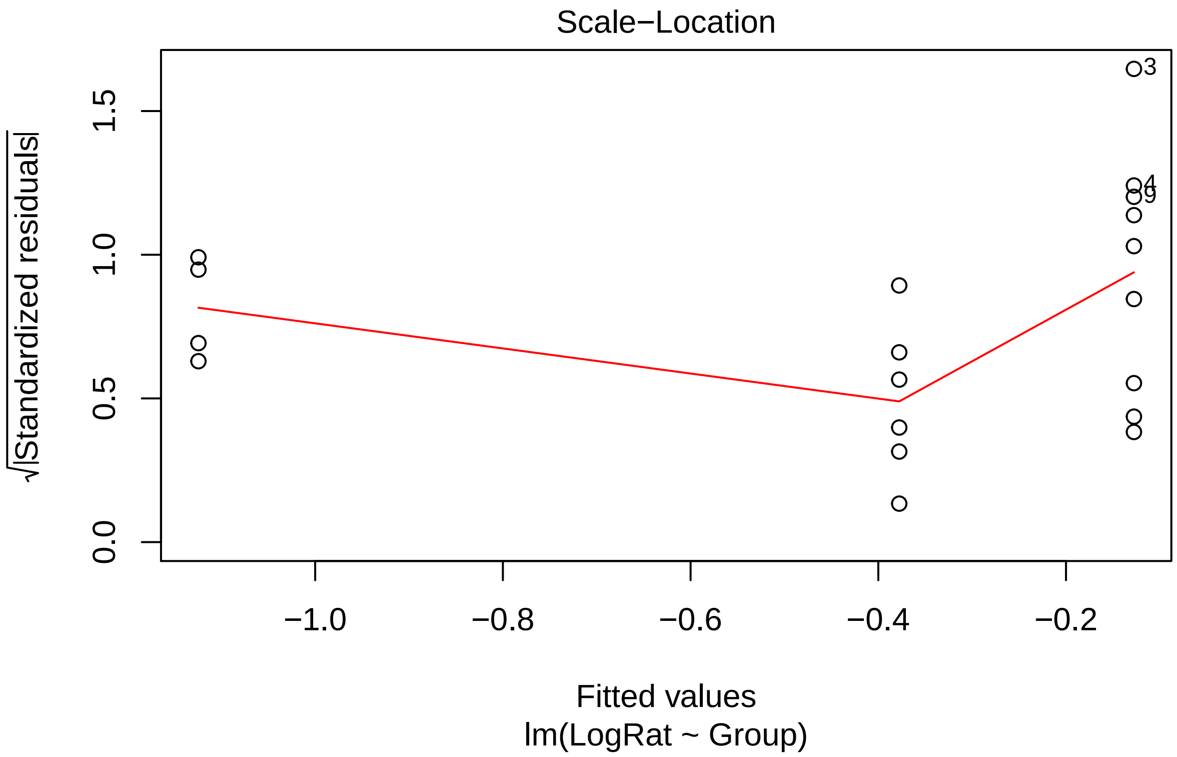
Scale−Location
√|Standardized residuals|
0.0
0.5
1.0
1.5
−1.0
−0.8
−0.6
−0.4
−0.2
3
4
9
Fitted values
lm(LogRat ~ Group)

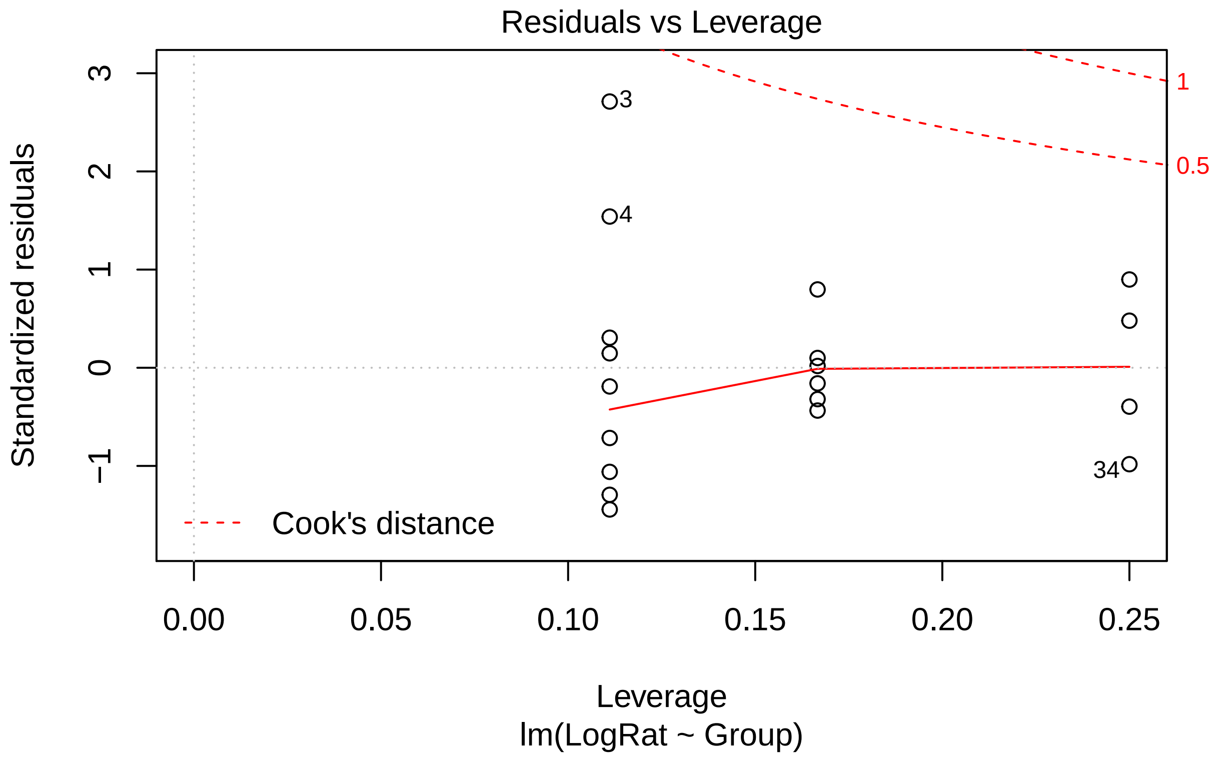

We see there are otlying data, and the distribution of residuals is not exactly Gaussian.

We will detect the outliers using a very impartial tool, then replace them with linear model predictions

```
## Loading required package: extremevalues

## [1] "Outliers detected:"

##   Location    Group Cycle       C Gene    Cref   LogRat
## 3       8E KONTROLA 27.87 3.53099 eNOS 1.44032 0.896713
## 4       8E KONTROLA 27.93 3.38676 eNOS 2.15049 0.454178

## [1] Location Group    Cycle    C        Gene     Cref     LogRat
## <0 rows> (or 0-length row.names)

## [1] "Replaced with expectations: Outliers"

##   Location    Group Cycle       C Gene    Cref LogRat Outlier LogRat.Orig
## 3       8E KONTROLA 27.87 3.53099 eNOS 1.44032     NA    TRUE    0.896713
## 4       8E KONTROLA 27.93 3.38676 eNOS 2.15049     NA    TRUE    0.454178

## Warning: Removed 2 rows containing missing values (geom_point).
```

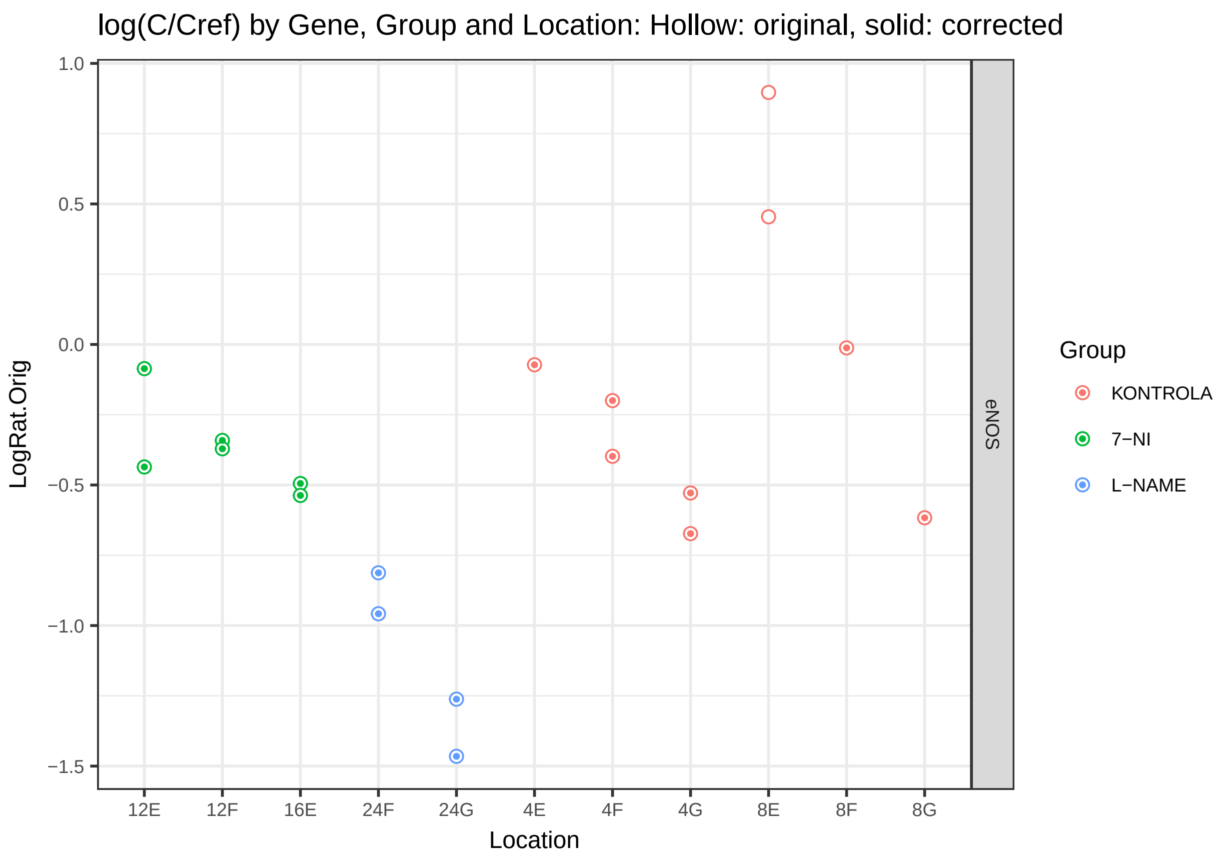

We detected 13 values (out of 306) and replaced them with linear fit predictions.

Fit linear model to data: log(C/Cref) ~ Gene : Group + Location

```
##
## Call:
## lm(formula = LogRat ~ Group, data = measurement)
##
## Residuals:
##     Min      1Q  Median      3Q     Max
## -0.3409 -0.1595 -0.0408  0.1664  0.3450
##
## Coefficients:
##             Estimate Std. Error t value Pr(>|t|)
## (Intercept) -0.35707    0.09103  -3.922 0.001533 **
## Group7-NI   -0.02062    0.13400  -0.154 0.879875
## GroupL-NAME -0.76733    0.15096  -5.083 0.000167 ***
## ---
## Signif. codes:  0 '***' 0.001 '**' 0.01 '*' 0.05 '.' 0.1 ' ' 1
##
## Residual standard error: 0.2409 on 14 degrees of freedom
##   (2 observations deleted due to missingness)
## Multiple R-squared:  0.684,  Adjusted R-squared:  0.6389
## F-statistic: 15.15 on 2 and 14 DF,  p-value: 0.0003146
```

We see that the model diagnostics are improved, but the residuals are still slightly non-gaussian.

ANOVA for the linear model

```
## Analysis of Variance Table
##
## Response: LogRat
##           Df  Sum Sq Mean Sq F value    Pr(>F)
## Group      2 1.75800 0.87900  15.152 0.0003146 ***
## Residuals 14 0.81214 0.05801
## ---
## Signif. codes:  0 '***' 0.001 '**' 0.01 '*' 0.05 '.' 0.1 ' ' 1
```

Express data as combinations of fit coefficients. Express group means as combinations of fit coefficients.

```
##       Group (Intercept) Group7-NI GroupL-NAME
## 1  KONTROLA           1         0           0
## 3  KONTROLA           1         0           0
## 4  KONTROLA           1         0           0
## 5  KONTROLA           1         0           0
## 6  KONTROLA           1         0           0
## 8  KONTROLA           1         0           0
## 9  KONTROLA           1         0           0
## 10 KONTROLA           1         0           0
## 12 KONTROLA           1         0           0
## 13     7-NI           1         1           0
## 14     7-NI           1         1           0
## 15     7-NI           1         1           0
## 16     7-NI           1         1           0
## 17     7-NI           1         1           0
## 18     7-NI           1         1           0
## 29   L-NAME           1         0           1
## 30   L-NAME           1         0           1
## 33   L-NAME           1         0           1
## 34   L-NAME           1         0           1
```

```
##      Group (Intercept) Group7-NI GroupL-NAME
## 1 KONTROLA           1         0           0
## 2     7-NI           1         1           0
## 3   L-NAME           1         0           1
```

Plot fitted data

```
## Warning: Removed 2 rows containing missing values (geom_point).

## Warning: Removed 2 rows containing missing values (geom_point).
```

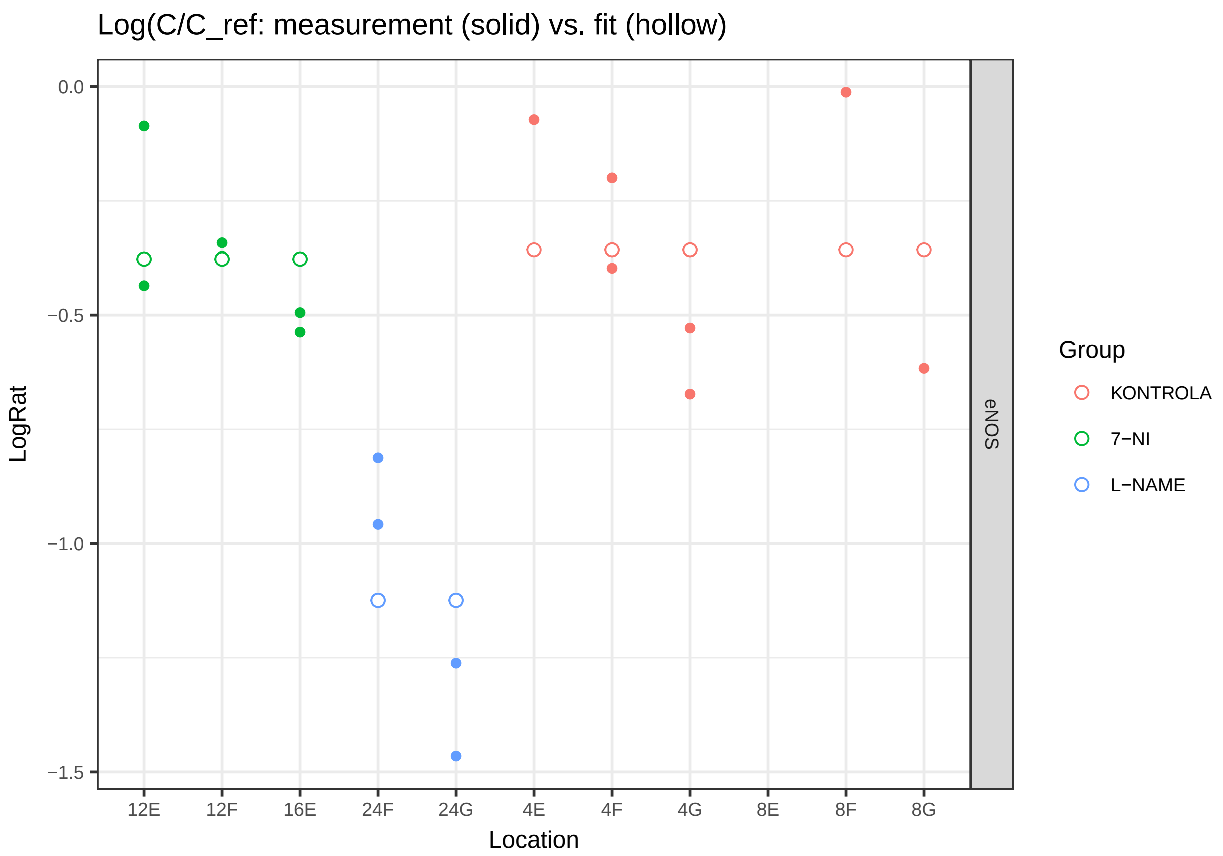

Multiple comparisons

Define the constrasts to test in terms of linear model coefficients.

```
##                           (Intercept) Group7-NI GroupL-NAME
## eNOS: 7-NI - KONTROLA               0         1           0
## eNOS: L-NAME - KONTROLA             0         0           1
## eNOS: L-NAME - 7-NI                 0        -1           1
```

```
## Loading required package: multcomp
```

```
## Loading required package: mvtnorm
```

```
## Loading required package: survival
```

```
## Loading required package: TH.data
```

```
## Loading required package: MASS
```

```
##
## Attaching package: 'TH.data'
```

```
## The following object is masked from 'package:MASS':
##
##     geyser
```

```
##
##   Simultaneous Tests for General Linear Hypotheses
##
```

```
## Fit: lm(formula = LogRat ~ Group, data = measurement)
##
## Linear Hypotheses:
##                              Estimate Std. Error t value Pr(>|t|)
## eNOS: 7-NI - KONTROLA == 0   -0.02062    0.13400  -0.154    0.987
## eNOS: L-NAME - KONTROLA == 0 -0.76733    0.15096  -5.083   <0.001 ***
## eNOS: L-NAME - 7-NI == 0     -0.74671    0.15547  -4.803   <0.001 ***
## ---
## Signif. codes:  0 '***' 0.001 '**' 0.01 '*' 0.05 '.' 0.1 ' ' 1
## (Adjusted p values reported -- single-step method)
```

The above are Bonferoni single-step adjusted p-values, that is, a very conservative estimate of significance.

Use Westfall-Young resampling to get more reasonable p-values

This is a much better method in that it is not dependent on Gaussianity and takes into account correlations between tests. It provides family-wise p-values, similar to the previous method.

```
##                  Contrast     t.stat p.adj
## 1   eNOS: 7-NI - KONTROLA -0.1539127 0.426
## 2 eNOS: L-NAME - KONTROLA -5.0829293 0.001
## 3     eNOS: L-NAME - 7-NI -4.8029105 0.001
```

We assemble the p-values from the previous methods to compare them.

Benjamini-Yekutieli FDR-adjusted p-values

This is a different method than the previous two in that it controls false discovery rate (FDR) rather than family-wise error. This is a method routinely used in very large molecular biology studies, where thousands of tests are performed simultaneously.

The B-Y method does not depend on correlations between tests and does not make any Gaussianity assumptions.

```
##           Method                Contrast        p.adj
## 1 Westfall-Young   eNOS: 7-NI - KONTROLA 0.4260000000
## 2 Westfall-Young eNOS: L-NAME - KONTROLA 0.0010000000
## 3 Westfall-Young     eNOS: L-NAME - 7-NI 0.0010000000
## 4      Bonferoni   eNOS: 7-NI - KONTROLA 0.9869884801
## 5      Bonferoni eNOS: L-NAME - KONTROLA 0.0004575464
## 6      Bonferoni     eNOS: L-NAME - 7-NI 0.0007769206
```

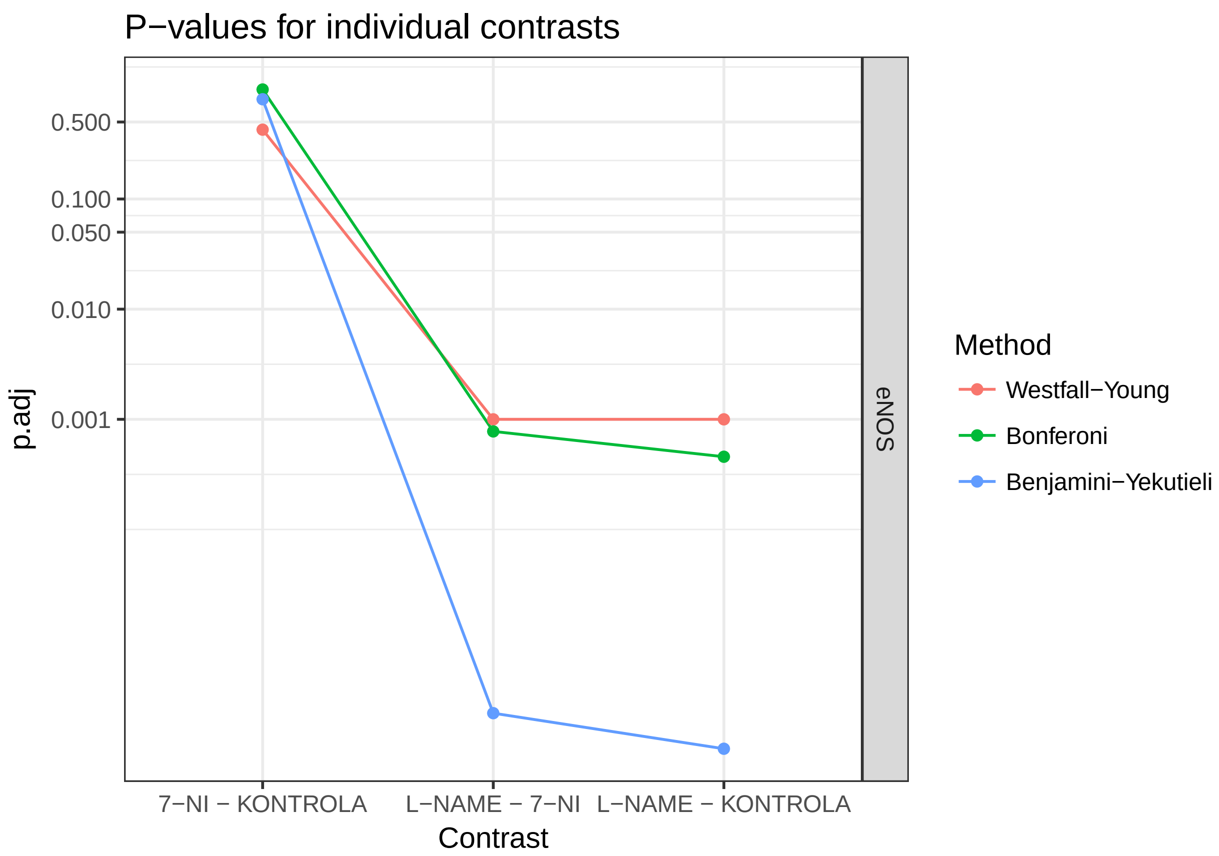

The minimum p-value the WY method can provide is limited by 1/(number of resamples), which is 0.0001 in our case. Therefore, WY does not follow the BY and Bonferoni values at low p's, while elsewhere the three p-values behave in a very simiilar manner, the Bonferoni p-values, as the most conservative, being the highest.

Apparently, B-Y can be used as a cheaper alternative to Westfall-Young, though the saving in simplicity is more important than that in computing time.

Plot contrasts

We plot the differences between groups that were tested above.

```
##                          Gene          Contrast    diff_log     sigma
## eNOS: 7-NI - KONTROLA    eNOS    7-NI - KONTROLA -0.02062401 0.1339981
## eNOS: L-NAME - KONTROLA eNOS L-NAME - KONTROLA -0.76733156 0.1509625
## eNOS: L-NAME - 7-NI      eNOS      L-NAME - 7-NI -0.74670754 0.1554698
##                               LegUp    LegDown
## eNOS: 7-NI - KONTROLA     0.1133741 -0.1546221
## eNOS: L-NAME - KONTROLA -0.6163691 -0.9182940
## eNOS: L-NAME - 7-NI     -0.5912377 -0.9021773
```

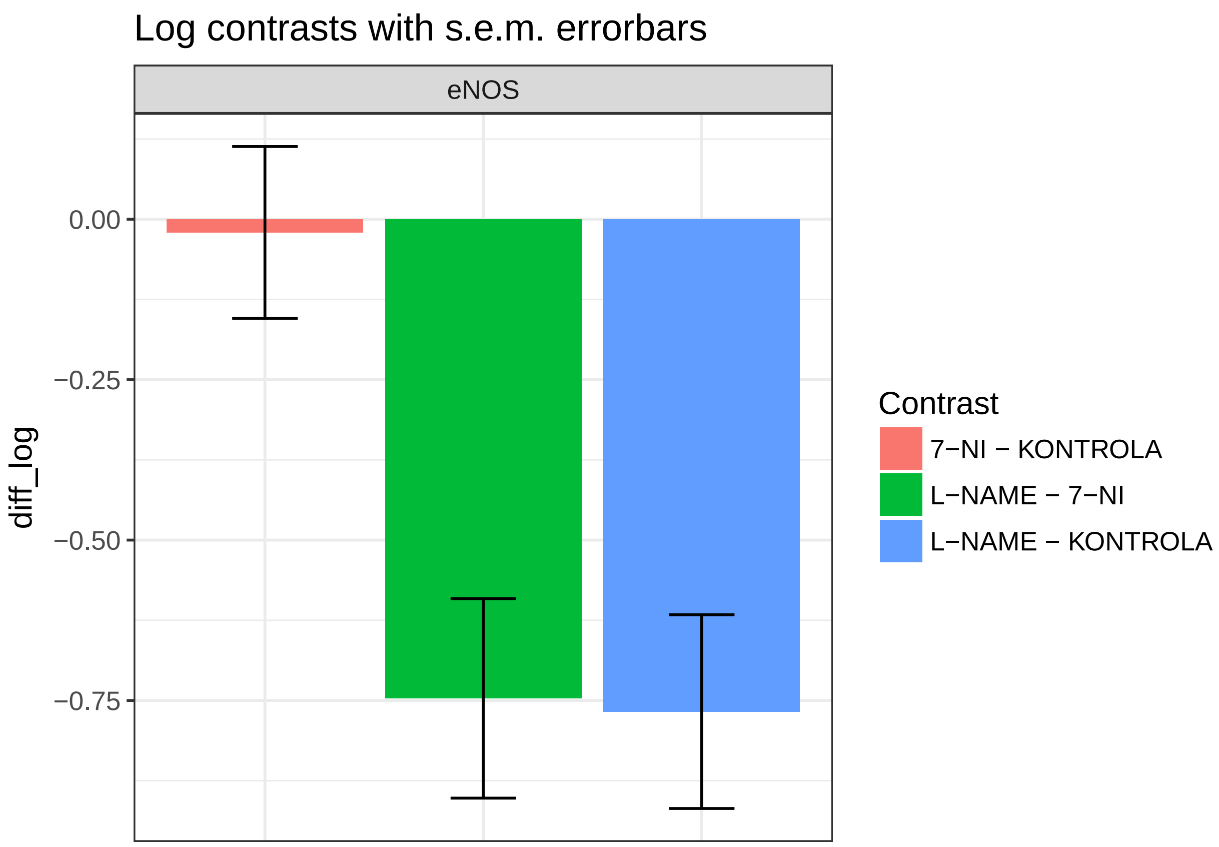

Finally, we plot gene/group means, which we should have done high above.

```
##      Group    LogRat      sigma     Ratio  LegDown     LegUp
## 1 KONTROLA -0.357071 0.09103379 0.6997228 0.6388378 0.7664106
## 2     7-NI -0.377695 0.09832774 0.6854395 0.6212494 0.7562621
## 3   L-NAME -1.124403 0.12042639 0.3248465 0.2879902 0.3664196
```

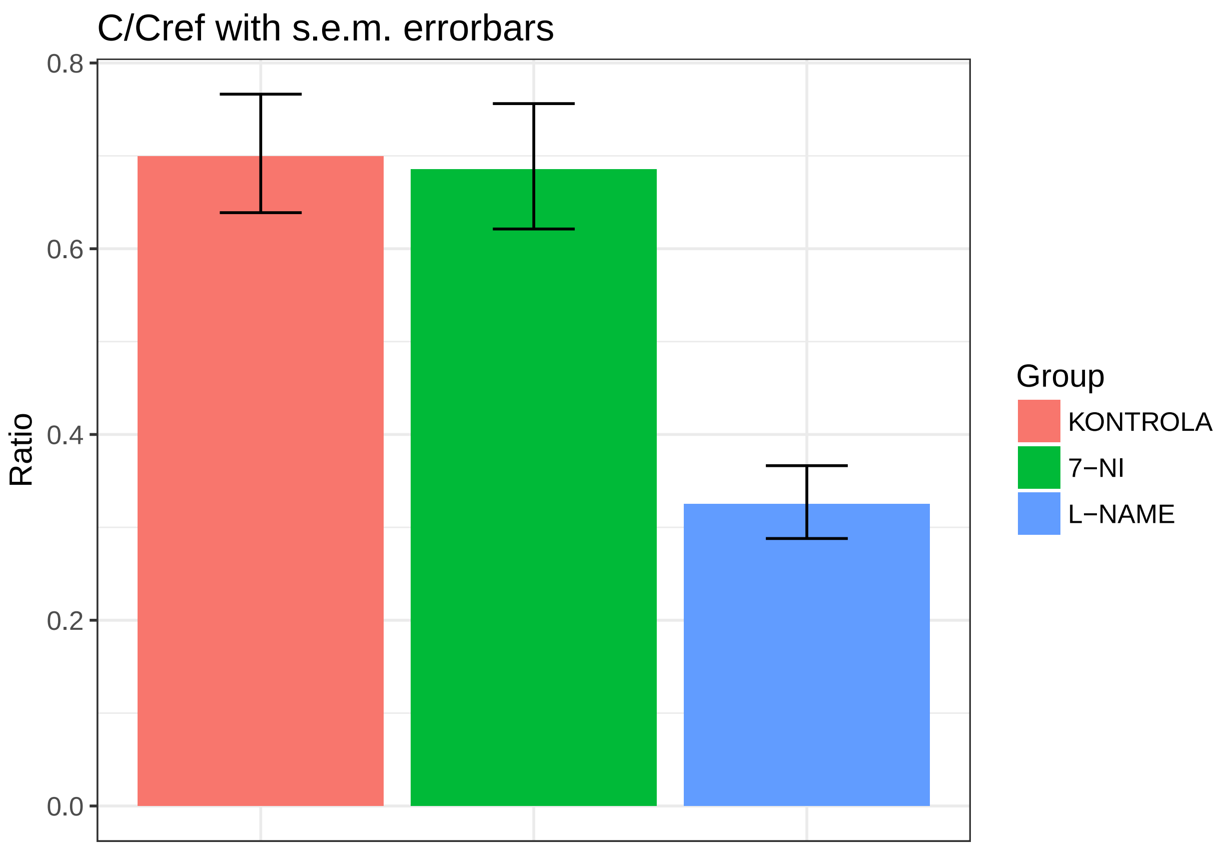


```
## Warning: Removed 2 rows containing non-finite values (stat_boxplot).

## notch went outside hinges. Try setting notch=FALSE.
## notch went outside hinges. Try setting notch=FALSE.
## notch went outside hinges. Try setting notch=FALSE.
```

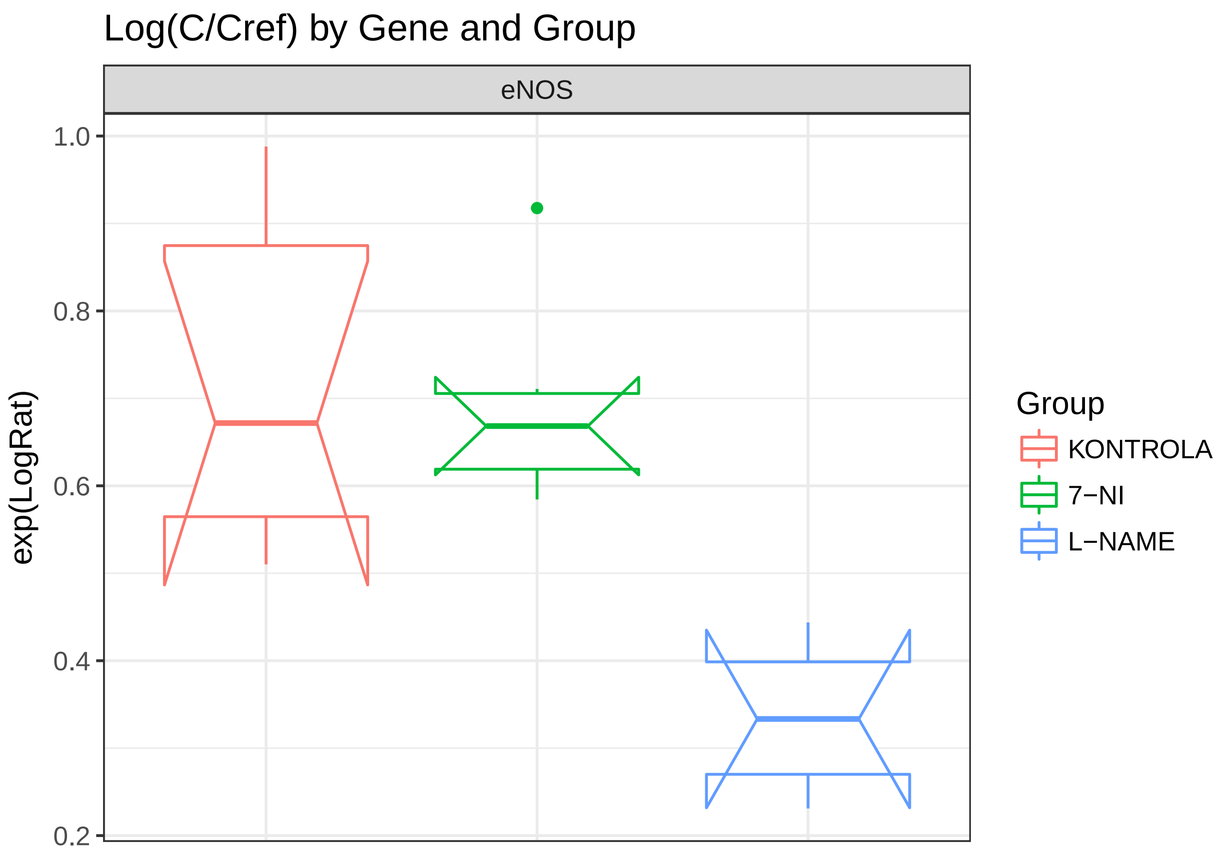
Log(C/Cref) by Gene and Group
eNOS
exp(LogRat)
1.0
0.8
0.6
0.4
0.2
Group
KONTROLA
7−NI
L−NAME

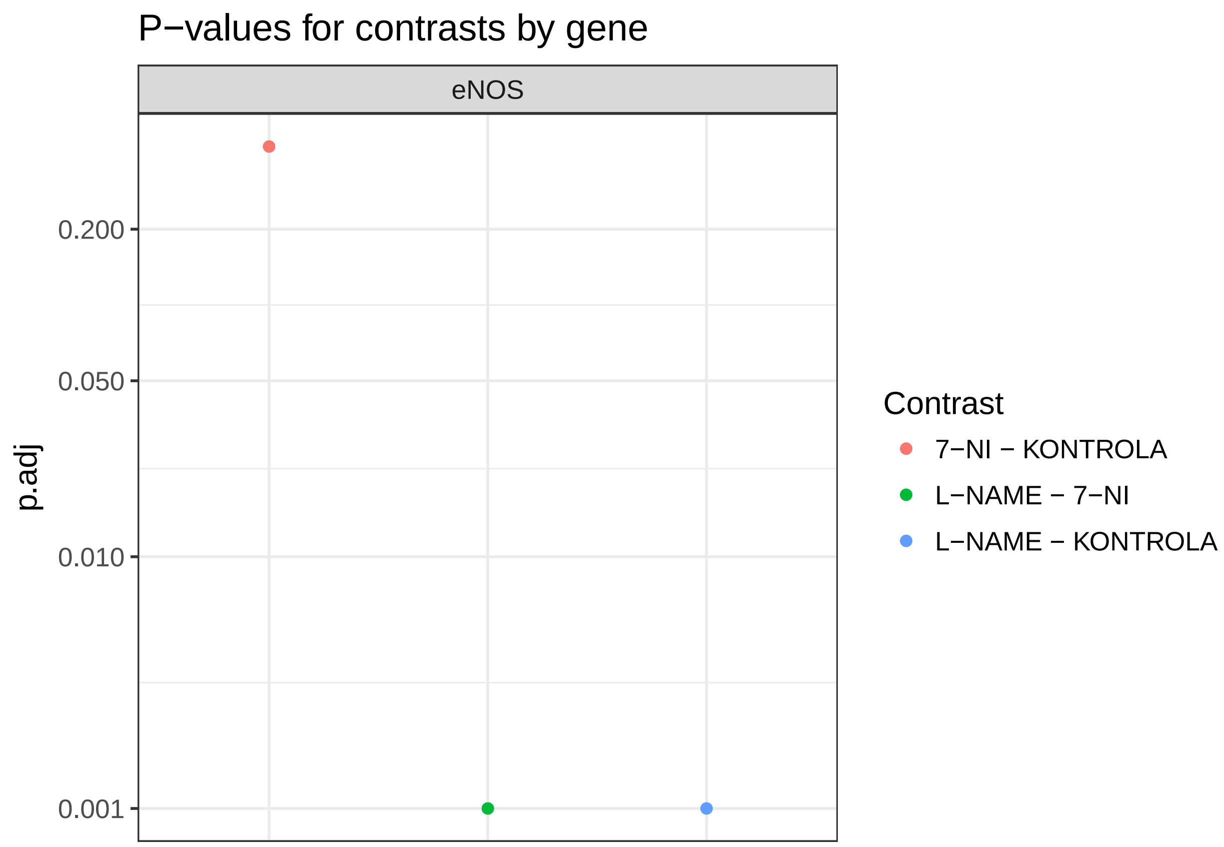


```
##               Method          Contrast         p.adj Gene
## 1      Westfall-Young   7-NI - KONTROLA 4.260000e-01 eNOS
## 2      Westfall-Young L-NAME - KONTROLA 1.000000e-03 eNOS
## 3      Westfall-Young     L-NAME - 7-NI 1.000000e-03 eNOS
## 4           Bonferoni   7-NI - KONTROLA 9.869885e-01 eNOS
## 5           Bonferoni L-NAME - KONTROLA 4.575464e-04 eNOS
## 6           Bonferoni     L-NAME - 7-NI 7.769206e-04 eNOS
## 7 Benjamini-Yekutieli   7-NI - KONTROLA 8.045387e-01 eNOS
## 8 Benjamini-Yekutieli L-NAME - KONTROLA 1.022060e-06 eNOS
## 9 Benjamini-Yekutieli     L-NAME - 7-NI 2.150167e-06 eNOS
```
